# The Role of Imaging Biomarkers to Guide Pharmacological Interventions Targeting Tumor Hypoxia

**DOI:** 10.3389/fphar.2022.853568

**Published:** 2022-07-15

**Authors:** Bernard Gallez

**Affiliations:** Biomedical Magnetic Resonance Research Group, Louvain Drug Research Institute, Université Catholique de Louvain (UCLouvain), Brussels, Belgium

**Keywords:** tumor hypoxia, oxygen, imaging, biomarker, cancer, predictive marker, therapy, theranostics

## Abstract

Hypoxia is a common feature of solid tumors that contributes to angiogenesis, invasiveness, metastasis, altered metabolism and genomic instability. As hypoxia is a major actor in tumor progression and resistance to radiotherapy, chemotherapy and immunotherapy, multiple approaches have emerged to target tumor hypoxia. It includes among others pharmacological interventions designed to alleviate tumor hypoxia at the time of radiation therapy, prodrugs that are selectively activated in hypoxic cells or inhibitors of molecular targets involved in hypoxic cell survival (i.e., hypoxia inducible factors HIFs, PI3K/AKT/mTOR pathway, unfolded protein response). While numerous strategies were successful in pre-clinical models, their translation in the clinical practice has been disappointing so far. This therapeutic failure often results from the absence of appropriate stratification of patients that could benefit from targeted interventions. Companion diagnostics may help at different levels of the research and development, and in matching a patient to a specific intervention targeting hypoxia. In this review, we discuss the relative merits of the existing hypoxia biomarkers, their current status and the challenges for their future validation as companion diagnostics adapted to the nature of the intervention.

## 1 At-A-Glance View of Tumor Hypoxia: Causes and Consequences

The overall goal of this manuscript is to provide the rationale for the development of companion diagnostics that are crucially important when developing and evaluating emerging hypoxia-targeted therapies. A companion diagnostic is a test (*in vitro* or *in vivo*) used to help match a patient to a specific drug or therapy. Before describing how imaging modalities could be helpful to guide hypoxia-targeted therapies, it is important to first briefly introduce the factors contributing to the occurrence of hypoxic regions in solid tumors as well as the cellular consequences of acute or prolonged periods of hypoxia. This will give a sense for understanding approaches aimed at alleviating hypoxia, on the one hand, and/or at fighting against downstream cellular responses at the origin of malignant progression and resistance to therapies, on the other hand.

### 1.1 Pathogenesis of Tumor Hypoxia

**FIGURE 1:**
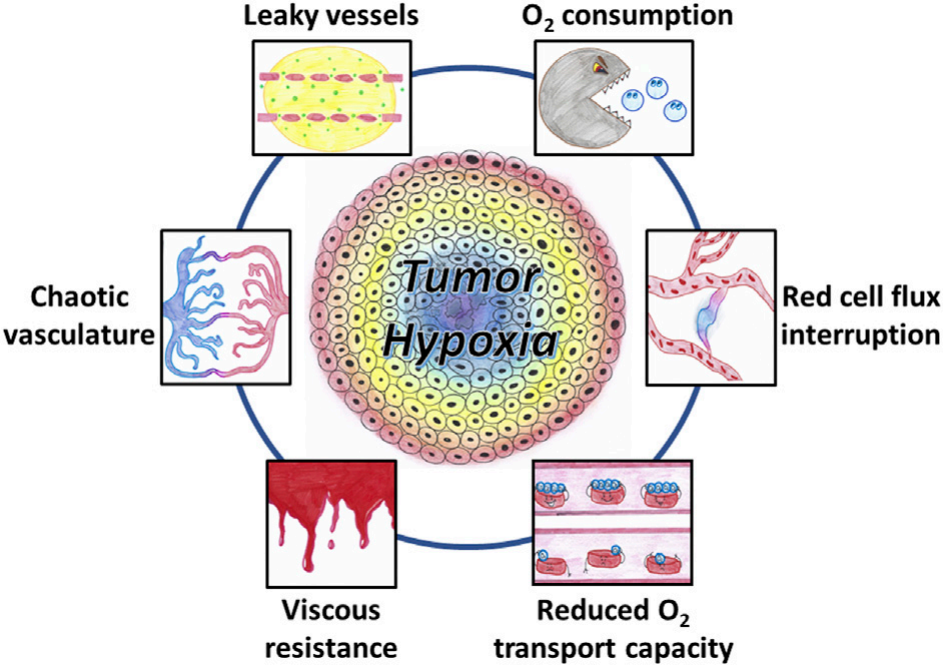
Factors contributing to the occurrence of tumor hypoxia. The tumor vasculature is chaotic, showing abnormal vascular density, contour irregularities, enlarged vessels, and vessels with blind ends. The increased leakiness of immature vessels results in an increased interstitial fluid pressure. Increased viscous resistance may contribute the vascular stasis. Tumor-associated anemia leads to a reduced oxygen transport capacity. Cycling hypoxia results from transient stasis in flow and transient interruption in red blood cell flux. Oxygen utilization by a large density of tumor cells with a high degree of metabolic activity and proliferation also contributes to tumor hypoxia.

The systematic detection of tumor hypoxia in the clinical setting has demonstrated that most solid tumors contain hypoxic regions that influence malignant progression and contribute to therapeutic resistance (Höckel and Vaupel, 2001; Vaupel et al., 2004; Vaupel et al., 2007; Vaupel and Mayer, 2007; Bayer et al., 2011; Busk and Horsman, 2013; Lee et al., 2014; Vaupel and Mayer, 2014; Horsman and Vaupel, 2016; Vaupel and Mayer, 2016; Hughes et al., 2019; Swartz et al., 2020; Sørensen and Horsman, 2020; Hompland et al., 2021; Vaup el et al., 2021). Mechanistically, tumor hypoxia is the result of an inadequate oxygen supply that cannot meet the oxygen demand by the cells present in the tumor microenvironment. Multiple factors contribute to the occurrence of tumor hypoxia (Figure 1) that exhibits a high degree of spatial and temporal heterogeneities (Dewhirst et al., 2008; Vaupel and Mayer, 2014; Vaupel and Mayer, 2016). While intrinsically related, the simplified view is to distinguish between chronic hypoxia and cycling hypoxia.

Chronic hypoxia (or diffusion-limited hypoxia) occurs with low-frequency variations (timeframe of hours, days or weeks) (Vaupel and Mayer, 2014; Vaupel and Mayer, 2016; Dewhirst et al., 2008). Chronic hypoxia is mainly caused by limitations in the diffusion of oxygen from the blood vessels to reach distant cells. Two types of oxygen gradient are existing in tumors as demonstrated by studies in window chamber tumors: a radial gradient due to enlarged diffusion distances from the perivascular space to distant cells, and a longitudinal gradient corresponding to a decline in vascular pO_2_ along the afferent path of blood flow (Dewhirst et al., 1994; Dewhirst et al., 1999). Several factors contribute to the occurrence of chronic hypoxia (Figure 1). Compared to the well-organized blood supply of normal tissues, the vascular system in tumors is chaotic. The tumor vascular supply shows abnormal vascular density, contour irregularities, enlarged vessels, and vessels with blind ends (Konerding et al., 1999; Horsman and Vaupel, 2016; Vaupel and Mayer, 2016; Sørensen and Horsman, 2020). In addition, the vessels originating from angiogenesis are often immature and highly permeable allowing significant plasma leakage. The increased leakiness and the absence of functional lymphatic drainage results in an increased interstitial fluid pressure leading to a decrease in pressure differences between arterial and venous ends causing blood flow stasis (Horsman and Vaupel, 2016). Moreover, increased viscous resistance may also contribute the vascular stasis. The lower pH resulting from metabolic adaptation to hypoxia and/or high glycolysis rate increases the rigidity of red blood cells and increases the blood viscosity (Jain, 1988; Sevick and Jain, 1989). Tumor-associated or therapy-induced anemia leads to a reduced oxygen transport capacity and can also contribute to the development of hypoxia (known as “anemic hypoxia” or “hypoxemic hypoxia”) (Vaupel and Mayer, 2007; Vaupel and Mayer, 2016).

Cycling hypoxia (or equivalently acute hypoxia or perfusion-limited hypoxia or fluctuating hypoxia or transient hypoxia) is the second major type of tumor hypoxia. Cycling hypoxia is characterized by episodes of hypoxia varying over shorter periods of time than chronic hypoxia (Brown, 1979; Chaplin et al., 1987; Dewhirst et al., 2008; Vaupel and Mayer, 2014). Experimental evidences demonstrated the occurrence of rapid cycles of fluctuating hypoxia (timeframe of a few seconds or less) and slow cycles (minutes to hours) (Braun et al., 1999; Braun et al., 2001; Baudelet et al., 2004; Baudelet et al., 2006; Cárdenas-Navia et al., 2008; Magat et al., 2010; Matsumoto et al., 2010; Yasui et al., 2010). Rapid cycles of hypoxia mainly result from transient stasis in flow or transient interruption in red blood cell flux (Dewhirst et al., 1996; Kimura et al., 1996) while it is speculated that slow cycles of hypoxia could be more related to vascular remodeling and presence of vascular smooth muscles (Baudelet et al., 2006; Bayer and Vaupel, 2012; Vaupel and Mayer, 2014; Bader et al., 2020).

While most factors described previously are related to the delivery of oxygen through the perfusion of the tumor, the oxygen utilization by cells present in the tumor microenvironment should not be neglected. First, solid tumors are composed of a large density of tumor cells with a high degree of metabolic activity and proliferation. Many tumor cells exhibit a glycolytic phenotype that provides a rapid production of ATP (much faster than mitochondria) and sustains cell proliferation through the pentose phosphate pathway. However, contrarily to the historical dogma, mitochondria remain functional in cancer cells that may exhibit a high respiratory capacity, and other substrates may fuel the electron transport chain (Zhdanov et al., 2014; De Preter et al., 2016a; Corbet and Feron, 2017; Marchetti et al., 2020; Vaupel and Multhoff, 2021a; Vaupel and Multhoff, 2021b). In addition, it has been shown that other cells present in the tumor microenvironment such as tumor associated macrophages (TAMs) present high oxidative phosphorylation with high basal and maximal oxygen consumption rate (M de-Brito et al., 2020). Overall, both impaired oxygen delivery and high oxygen cellular metabolic demand contribute to the prevalence of hypoxia in solid tumors.

### 1.2 Significance of Tumor Hypoxia

Experimental and clinical studies support the fact that hypoxia has detrimental consequences for both cancer progression and response to therapies.

#### 1.2.1 Cellular Response to Hypoxia

**FIGURE 2:**
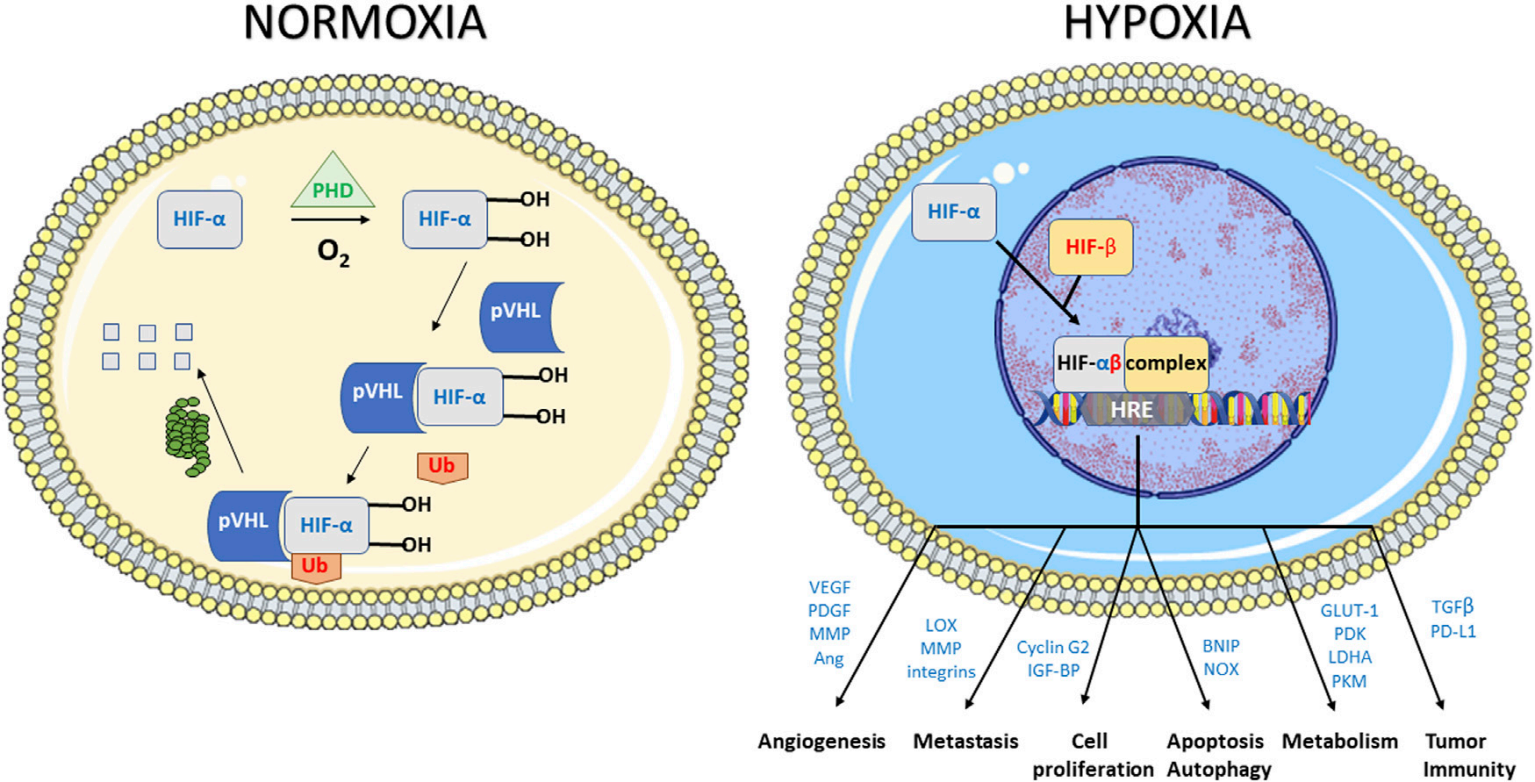
Response to hypoxic stress mediated by the hypoxia inducible factors (HIFs). HIF1s are heterodimeric proteins that consist of two proteins, HIF1-α and HIF1-β. In normoxia, HIF1-α undergoes proteasomal degradation by a mechanism that involves hydroxylation of proline residues on HIF1-α by prolyl hydroxylases (PHDs) and subsequent ubiquitination by the pVHL (von Hippel Lindau) system. In hypoxia, the PHDs lose their activity, the hydroxylation of the HIF1-α subunit is inhibited without subsequent degradation. The non-hydroxylated, stabilized HIF1-α subunits translocate to the nucleus where they dimerize with constitutively expressed HIF1-β subunit, and bind to DNA to initiate gene transcription. Illustrative genes that are transcriptionally activated by HIF-1 included those involved in angiogenesis, invasion and metastasis cell proliferation, apoptosis and autophagy, metabolism and tumor immunity.

In response to the stress caused by hypoxia, cells undergo a large variety of molecular responses (Harris, 2002; Vaupel and Mayer, 2016; Lee et al., 2020; Sørensen and Horsman, 2020). The predominant hypoxia-mediated intracellular signaling pathway is controlled by a family of transcription factors, the hypoxia inducible factors (HIFs) (Semenza and Wang, 1992; Wang and Semenza, 1993; Semenza, 2000; Harris, 2002; Semenza, 2003; Muz et al., 2015; Vaupel and Mayer, 2016; Pugh and Ratcliffe, 2017; Lee et al., 2020) (Figure 2). HIFs are heterodimeric proteins that consist of two proteins, HIF-α and HIF-β. HIF-α stability is the principal key factor for the regulation of HIF activity. HIF-α has three closely related homologues, HIF-1α, HIF-2α, and HIF-3α (Semenza, 2003; Loboda et al., 2010; Muz et al., 2015; Albadari et al., 2019; Codony and Tavassoli, 2021). In normoxia, HIF-α undergoes proteasomal degradation by a mechanism that involves hydroxylation of proline residues on HIF-α by prolyl hydroxylases (PHDs) and subsequent ubiquitination by the pVHL (von Hippel Lindau) protein E3 ubiquitin ligase system. In hypoxia, the PHDs lose their activity, the hydroxylation of the HIF-α subunit is inhibited without subsequent degradation. The non-hydroxylated, stabilized HIF-α subunits translocate to the nucleus where they dimerize with constitutively expressed HIF-β subunit, and bind to DNA to initiate gene transcription (Semenza, 2003; Loboda et al., 2010; Muz et al., 2015; Albadari et al., 2019; Codony and Tavassoli, 2021). Of note, the expression of HIF-1α could also be achieved in a hypoxia-independent manner, including by ROS and by growth factors through receptor tyrosine kinases (Semenza, 2003; Muz et al., 2015). The oxygen-independent HIF regulation is mediated by several signaling pathways including NFκB, PI3K/AKT/mTOR, and MAPK/ERK. These pathways are additionally regulated by hypoxia, which results in multiple levels of HIF-α stimulation, both hypoxic and normoxic (Harris, 2002; Semenza, 2003; Muz et al., 2015; Lee et al., 2020). Genes that are involved in tumor progression are transcriptionally activated by HIF-1 (Figure 2). Among them, target genes included those involved in angiogenesis (VEGF, VEGF-R1, VEGF-R2, PDGF, Ang-1, Ang-2, MMPs), invasion and metastasis (LOX, MMPs, integrins), epithelial-to-mesenchymal transition (EMT) modulation (cadherins, vimentin), cell proliferation (cyclin G2, IGF-BPs), cell survival (ADM, IGF2, IGF-BPs, TGF-β), apoptosis and autophagy (BNIP, NOX), metabolism (GLUT1, GAPDH, PDK, LDHA,PKM), regulation of tumor acidosis (CAIX), and tumor immunity (TGF-β, PD-L1) (Semenza, 2000; Harris, 2002; Feldman et al., 2005; Horsman and Vaupel, 2016; Lee et al., 2020; Codony and Tavassoli, 2021). It should be emphasized that this list of target genes is illustrative rather than exhaustive.

Under severe hypoxia, one of the stress responses (HIF-independent) is the unfolded protein response (UPR) activated in response to ER (endoplasmic reticulum) stress (Feldman et al., 2005; Horsman and Vaupel, 2016; Lee et al., 2020). ER stress induces cytoprotective functions by activating signaling pathways to keep cellular homeostasis. However, if the stress remains unresolved, signaling pathways will activate apoptosis. The UPR is mediated through the activation of ER transmembrane stress sensors: pancreatic ER kinase (PKR)-like ER kinase (PERK), activating transcription factor 6 (ATF6) and inositol-requiring enzyme 1 (IRE1). These proteins are in an inactive state through a physical interaction between their ER lumen domains and GRP78 (a chaperone glucose-regulated protein of 78 kDa). If the level of unfolded proteins increases in the ER, GRP78 will be redirected to these unfolded proteins, with a release and activation of the ER stress sensors, launching the UPR (Horsman and Vaupel, 2016).

Differential consequences can be actually observed depending on the cell type exposed, the degree of hypoxia and the exposure time to hypoxia (Michiels et al., 2016; Vaupel and Mayer, 2016). Both chronic and acute hypoxia may foster tumor progression. However, it has been suggested in preclinical tumor models that cycling hypoxia through specific signaling pathways, genomic instability and enhanced ROS production may lead to even greater tumor aggressiveness (Cairns et al., 2001; Cairns and Hill, 2004; Dewhirst et al., 2008; Martinive et al., 2009; Miao et al., 2014; Michiels et al., 2016; Vaupel and Mayer, 2016).

#### 1.2.2 Hypoxia as Factor of Resistance to Therapy

While long-term exposure to severe hypoxic conditions is lethal for many cells, subpopulations of tumor cells could adapt to hypoxic conditions and become resistant to radiotherapy, chemotherapy and immunotherapy (Höckel and Vaupel, 2001; Cosse and Michiels, 2008; Rohwer and Cramer, 2011; Muz et al., 2015).

**FIGURE 3:**
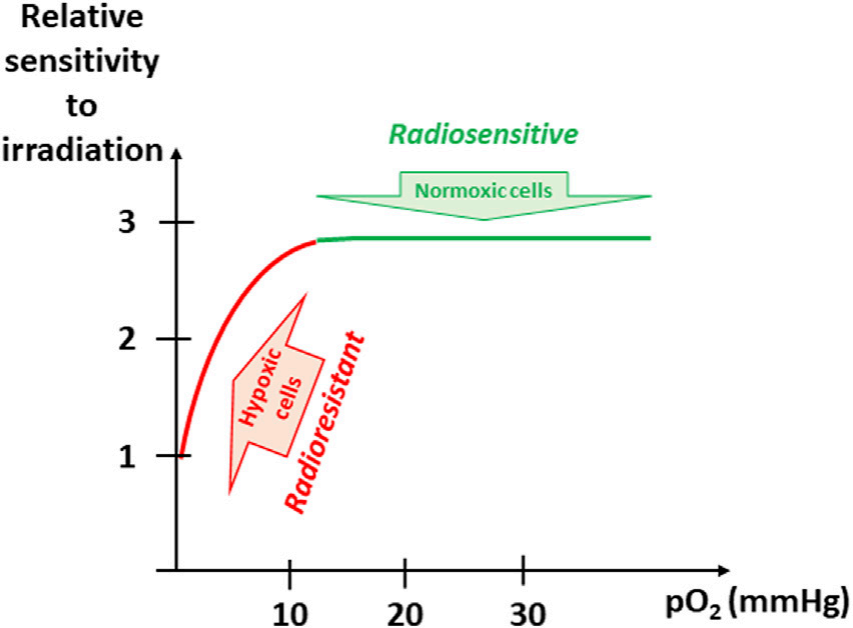
Evolution of sensitivity to irradiation as a function of pO_2_. The “oxygen enhancement ratio” (OER, the ratio of doses required to obtain the same cell survival under hypoxic and aerobic conditions) varies from 2.5 to 3.0, indicating that hypoxic tumor cells will require a dose 2.5–3 times higher to be killed than normoxic cells. The OER is dramatically increasing when pO_2_ is rising from 1 to 10 mmHg (found in hypoxic tumors). Above this value of 10 mmHg, further increase in pO_2_ does not further enhance the radiosensitivity.

One of the most studied resistance to therapy linked to tumor hypoxia is the resistance to radiation therapy. The potential involvement of oxygen as a modulator of response to irradiation was published already more than 100 years ago. G. Schwarz described the effect of hypoxia as protector from radiation (Schwarz, 1909). He observed that skin compression and reduction in skin blood flow decreased the radiosensitivity (actually reduced radiation burn) (Schwarz, 1909). About 70 years ago, the seminal studies of L.H. Gray and R.H. Thomlinson suggested the importance of microregional structures and associated oxygen gradient for the response to irradiation (Gray et al., 1953; Thomlinson and Gray, 1955). Since these early studies, several thousands of manuscripts have been published on this thematic research [on 28 December 2021, 8,321 references found in Pubmed for a research associating (hypoxia) and (radiation)]. The mechanism responsible for the enhancement of radiation damage by oxygen is generally referred to as the oxygen-fixation hypothesis (Horsman et al., 2009). DNA is generally considered as the ultimate target leading to a mitotic catastrophe (clonogenic death). Damages to DNA may be produced directly or indirectly through the water radiolysis and production of highly reactive free radicals which ultimately produced in DNA a transient radical R^•^ from RH. When oxygen is present, R^•^ can immediately react with O_2_ to produce ROO^•^ to further produce ROOH. In other words, oxygen is “fixing” the DNA damage with a change in the chemical structure of DNA. In the absence of oxygen, the unstable R^•^ molecules have a longer half-life and can react with H^•^, thus chemically restoring their original form (Horsman et al., 2009). The “oxygen enhancement ratio” (OER, the ratio of doses required to obtain the same cell survival under hypoxic and aerobic conditions) varies from 2.5 to 3.0, indicating that hypoxic tumor cells will require a dose 2.5–3 times higher to be killed than normoxic cells (Brown, 2007; Horsman et al., 2009). From literature, it appears that OER is dramatically increasing when pO_2_ is rising from 1 to 10 mmHg (Whillans and Hunt, 1982; Koch et al., 1984; Wouters and Brown, 1997; Brown, 2007; Horsman et al., 2009). Above this value of 10 mmHg, further increase in pO_2_ does not further enhance the radiosensitivity (Figure 3). The effect of tumor hypoxia on the response to a treatment by ionizing radiation has been demonstrated in a multitude of experimental preclinical studies [reviewed in (Vaup el et al., 2021) and (Gallez, 2021)]. In a series of clinical studies in the early nineties, using pO_2_ measurements with microelectrodes, P. Vaupel and others definitively demonstrated that tumor hypoxia was predicting the response of tumors to radiation therapy (Gatenby et al., 1988; Höckel et al., 1993a; Höckel et al., 1993b; Okunieff et al., 1993; Stone et al., 1993; Höckel et al., 1996; Fyles et al., 1998; Knocke et al., 1999; Höckel and Vaupel, 2001; Rudat et al., 2001; Nordsmark et al., 2005; Vaupel et al., 2007; Vaupel and Mayer, 2007; Vaup el et al., 2021). Although tumor hypoxia is acknowledged as the major factor of resistance of solid tumors to radiation therapy, the clinical practice actually does not yet fully integrate on a routine basis this factor in the definition of radiation protocols. Possible strategies for improving the curative effect of radiotherapy on hypoxic cells include the alleviation of tumor hypoxia at the time of irradiation and/or the redistribution of the radiation dose integrating the presence of hypoxic areas. From a meta-analysis gathering 10,108 patients from 86 randomized trials designed to modify tumor hypoxia in patients treated with curative attempted primary radiation (Overgaard, 2007), J. Overgaard concluded that “*Ample data exist to support a high level of evidence for the benefit of hypoxic modification. However, hypoxic modification still has no impact on general clinical practice*” (Overgaard, 2007). In a second meta-analysis, he analyzed the results from clinical trials that included 4,805 patients suffering from squamous cell carcinoma of the head and neck (HNSCC) with attempts to modify the hypoxic radioresistance (by applying normobaric oxygen, carbogen breathing, hyperbaric oxygen or hypoxic radiosensitizers) (Overgaard, 2011). Again, he demonstrated the added value of adding hypoxic modification to radiotherapy in HNSCC patients (Overgaard, 2011). In discussing the results from these meta-analyses, J. Overgaard pointed that the lack of proper identification of the patients with hypoxic tumors requiring adapted treatment was an obstacle to a routine clinical use of hypoxia-targeted interventions (Overgaard, 2007).

Tumor hypoxia is also very likely a key detrimental factor for the resistance to anti-cancer chemotherapy. Contrarily to the clinical evidence existing in the field of radiation therapy, there is no direct existing clinical proof linking the level of tumor hypoxia and the (absence of) response to specific chemotherapeutic agent. This is due to the fact that the use of chemotherapy for treating solid tumors is always part of a combined strategy together with surgery and/or radiation therapy without possibility to isolate the role of hypoxia on the sole drug response. However, a compelling body of experimental evidence demonstrates that hypoxia may alter the response to different chemotherapeutic agents. We have already discussed that HIF-mediated cellular processes may alter cell apoptosis, autophagy and tumor stemness which can have a direct impact on the drug response (Cosse and Michiels, 2008; Das et al., 2008; Rohwer and Cramer, 2011). In addition, tumor cells divide at a reduced rate as a result of decline in nutrient and oxygen availability. Consequently, the effect of drugs whose activity is selective for rapidly dividing cell populations is decreased. A large difference in proliferation rate between cells located in perivascular areas and those adjacent to necrotic regions has been clearly demonstrated (Tannock, 1968; Olive, 1989). In addition to hypoxia-induced cellular adaptations, it has been demonstrated that low oxygen level may alter the response to platinum complexes (Fadejeva et al., 2017), doxorubicin (Frederiksen et al., 2003), etoposide (Cosse et al., 2007; Cosse et al., 2009; Sermeus et al., 2013), bleomycin (Roizin-Towle and Hall, 1979). Of note, hypoxia may also affect the expression and activity of drug efflux pump such as p-glycoprotein (P-gp) and therefore contribute to a lower intracellular concentration of active drug (Thews et al., 2008; Abraham et al., 2015). Finally, considering that tumor hypoxia is associated with an abnormal vascularization, the impaired delivery of drugs also contributes as mechanism of resistance to the response to chemotherapy (Jain, 1991; Eskey et al., 1994; Netti et al., 1995; Jain, 1996; Jain, 1997; Tong et al., 2004; Ansiaux et al., 2006a; Martinive et al., 2006; Segers et al., 2006; Cron et al., 2008; Goel et al., 2011; Chauhan et al., 2013).

Tumor hypoxia is also a critical factor involved in the response to immunotherapy through multiple mechanisms (Noman et al., 2015; Lequeux et al., 2019; Noman et al., 2019; Chouaib, 2020; Multhoff and Vaupel, 2020; Terry et al., 2020; Fu et al., 2021; You et al., 2021). Low oxygen tension in tumors may act by reducing survival, cytolytic and migratory activity of immunostimulatory effector cells such as CD4^+^ cells, CD8^+^ cytotoxic T cells, natural killer-like T (NKT) cells and natural killer (NK) cells (Kumar and Gabrilovich, 2014; Samanta and Semenza, 2018; Multhoff and Vaupel, 2020). The stabilization of HIF-1α upregulates the expression of Programmed death-ligand 1 (PD-L1) in hypoxic tumor cells as well as the immune checkpoint V-Domain Ig suppressor of T cell activation (VISTA) in hypoxic myeloid-derived suppressor cells (MDSCs). The increased expression of PD-L1 and VISTA results in an inhibition of T cell proliferation and T cell mediated lysis (No man et al., 2014; Deng et al., 2019; Noman et al., 2019). HIF-1α is also involved in the upregulation of the macrophage immune checkpoint CD47 on the surface of tumor cells inducing tumor cell escape from phagocytosis (Noman et al., 2015; Zhang et al., 2015). Hypoxia-induced autophagy also impairs tumor cell susceptibility to CTL and NK-mediated lysis (Viry et al., 2014; Noman et al., 2015). Finally, hypoxia upregulates the expression of immunosuppressive HLA-G on the surface of tumor cells. The immunosuppressive functions of HLA-G depend on the binding to ILT2, ILT4, and KIR2DL4 expressed by several immune cells, including B cells, T cells, NK cells, dendritic cells, monocytes, and macrophages. As a consequence, the hypoxic-dependent overexpression of HLA-G also contributes to tumor escape from immune surveillance (Noman et al., 2015; Garziera et al., 2017). This hypoxia-mediated immunosuppression has stimulated the research for interventions to improve immunotherapy responsiveness, including alleviation of tumor hypoxia, the use of hypoxia-activated drugs and HIF inhibitors (Noman et al., 2015; Fu et al., 2021).

## 2 Treatments Targeting Hypoxia

The treatments targeting tumor hypoxia can be classified in three main categories: 1) attempts to alleviate tumor hypoxia in order to optimize the response to radiation therapy; 2) prodrugs that are activated to become toxic selectively in hypoxic cells; 3) inhibitors of molecular targets involved in hypoxic cell survival. It should be emphasized that these strategies have been tested mostly in preclinical models. The attempts that have been translated into the clinic are also compiled in the next sections.

### 2.1 Alleviation of Tumor Hypoxia at the Time of Radiation Therapy

As most solid tumors contain hypoxic regions that can be resistant to irradiation, the alleviation of tumor hypoxia could lead to a therapeutic benefit when combined with radiation therapy. A transient increase in tumor oxygenation at the time of irradiation is generally considered as a safe approach because it will directly impact the radioresistant hypoxic (tumor) cells without affecting the well oxygenated (normal) tissues (Figure 3). It has been described that tumor hypoxia may be considerably influenced by “physical treatments.” The most known example relies on the early changes in oxygenation observed after irradiation itself. The hypothesis of a tumor reoxygenation has been established several decades ago as part of the rule of the 4 *Rs* or 6 *Rs* (Radiosensitivity, Repair, Repopulation, Redistribution, Reoxygenation, and Reactivation of anti-tumor immune response) describing the response to an irradiation (Rakotomalala et al., 2021). Experimental evidences with quantitative and dynamic assessments of tumor oxygenation have demonstrated the occurrence of these effects and their contributing factors suggesting that appropriate scheduling may be exploited to potentiate the efficacy of radiation therapy (Olive, 1994; Goda et al., 1995; O'Hara et al., 1995; Goda et al., 1996; O'Hara et al., 1998; Sonveaux et al., 2002; Crokart et al., 2005a; Cron et al., 2005; Hou et al., 2013). Modulations in tumor oxygenation have been also observed after change in temperature (hyper- or hypothermia) (Moon et al., 2010; Neveu et al., 2017) or application of photodynamic therapy using verteporfin or redaporfin as photosensitizer (Pogue et al., 2002; Pogue et al., 2003; Karwicka et al., 2019). Another efficient method to increase tumor oxygenation is to provide a gas enriched in oxygen, for example 100% oxygen or carbogen (i.e., mixture of 95% O_2_ and 5% CO_2_ or 98% O_2_ and 2% CO_2_) (Siemann et al., 1977; Rojas et al., 1990; Falk et al., 1992; Grau et al., 1992; Horsman et al., 1994; Hoskin et al., 1997; Hill et al., 1998; Kaanders et al., 1998; Powell et al., 1999; Kaanders et al., 2002; Overgaard, 2007; Hoskin et al., 2009; Khan et al., 2009; Hoskin et al., 2010; Khan et al., 2010; Overgaard, 2011; Janssens et al., 2012; Thews and Vaupel, 2016; Song et al., 2021). Hyperbaric oxygen has also demonstrated a beneficial effect to reoxygenate tumors (Henk et al., 1977; Haffty et al., 1999; Becker et al., 2002; Thews and Vaupel, 2015; Chen et al., 2021). While breathing oxygen or carbogen is efficient in alleviating tumor hypoxia, some pharmacological approaches have led to a better response in sensitizing tumors to irradiation because they may act by several mechanisms including intrinsic radiosensitizing properties.

**FIGURE 4:**
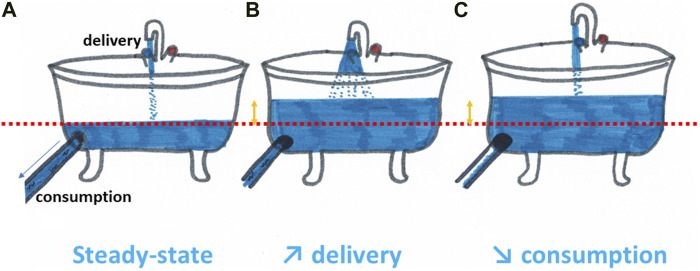
Increasing tumor oxygenation can be compared with the filling of a bath. To rise the water level in a bathtub compared to the steady state **(A)**, you may either increase the water supply by playing with the faucet **(B)** or decrease the opening of the draining plug **(C)**. In a similar manner, pharmacological strategies aimed at increasing the tumor oxygenation are targeting either the oxygen delivery (through an increase in perfusion, a decrease in blood viscosity or a better release of oxygen from hemoglobin) or the oxygen consumption (through the decrease of metabolic activity of the tumor cells).

Most attempts of alleviation of tumor hypoxia by pharmacological agents have been assessed in the context of improving the response to irradiation. Conceptually, the strategies to increase the tumor oxygenation are comparable with the filling of a bath. To rise the water level in a bathtub, you may either increase the water supply by playing with the faucet or decrease the opening of the draining plug (Figure 4). In a similar manner, pharmacological strategies aimed at increasing the tumor oxygenation are targeting either the oxygen delivery (through an increase in perfusion, a decrease in blood viscosity or a better release of oxygen from hemoglobin) or the oxygen consumption (through the decrease of metabolic activity of the tumor cells).

#### 2.1.1 Decreasing Tumor Hypoxia by Improving the Oxygen Delivery

Historically, it has been thought that increasing the perfusion (and consequently the oxygenation) could be elusive because of the lack of autoregulation by tumor blood vessels. Indeed, immature vessels derived from the angiogenic processes do not function as normal contractile cells (Jain, 1988; Lübbe and Huhnt, 1994; Eberhard et al., 2000; Sonveaux, 2008). However, the tumor vasculature also contains vessels that are able to regulate blood flow (Lübbe and Huhnt, 1994; Bergers and Benjamin, 2003; Julien et al., 2004; Sonveaux, 2008), including coopted preexisting vessels with contractile properties, vessels closed to the tumor margin, and vessels that gain structural maturity to acquire vasoactive capabilities (Peterson and Matts on, 1984; Sonveaux, 2008). Agents acting on the vasomotion of these vessels could therefore be used to increase blood flow and oxygenation. Thanks to methods that allow longitudinal quantitative measurements of tumor oxygenation such as Electron Paramagnetic Resonance (EPR) oximetry (Gallez et al., 2004; Khan et al., 2007; Ahmad and Kuppusamy, 2010), experimental evidences have shown the validity of such approaches. In a very large screening among 34 vasoactive agents (including angiotensin-converting enzyme inhibitors, alpha antagonists, beta-blockers, potassium channel openers, calcium antagonists, NO donors, and peripheral vasoactive agents), it was found that 24 compounds induced a significant increase in tumor oxygenation (Gallez et al., 1999a). Several compounds had profound effect on tumor oxygenation status and were further characterized for their effect on tumor hemodynamics and potential radiosensitizing properties (Gallez, 2021). The increase in tumor oxygenation observed after pre-treatment with nicotinate derivatives (xanthinol nicotinate or benzylnicotinate) and nitrosocaptopril significantly increased the tumor response to irradiation (Hou et al., 2010; Jordan et al., 2010; Segers et al., 2010). It was also found that diphteria toxin decreased the interstitial fluid pressure in solid tumors (Padera et al., 2004). The local administration of botulin neurotoxin was also found to significantly increase the tumor perfusion and oxygenation through an inhibition of neurotransmitter release and neurogenic contraction (Ansiaux et al., 2006a; Ansiaux and Gallez, 2007; Cron et al., 2008). The opening of the vascular bed induced by the local delivery of botulin neurotoxin led to an increase in the response of tumors to radio- and chemotherapy (Ansiaux et al., 2006a; Cron et al., 2008). It was also described that the endothelin receptor A (ETA) antagonist BQ123 decreased the vascular tone of tumor arterioles and increased tumor perfusion and oxygenation with a consequent improved response to radiation therapy and chemotherapy (Sonveaux et al., 2004; Martinive et al., 2006). Still, the success of these approaches playing on the vascular tone will definitely depend on the proportion of vasoreactive vessels compared to immature vessels present in the tumors. In this regard, the ability to measure the impact of those treatments on tumor perfusion and oxygenation by functional imaging will be crucial for a successful personalized treatment.

Another concept that has received particular intention is the normalization of tumor vasculature at the early phase of antiangiogenic treatments (Jain, 2001; Tong et al., 2004; Ansiaux et al., 2005; Jain, 2005; Segers et al., 2006; Batchelor et al., 2007; Mazzone et al., 2009; Carmeliet and Jain, 2011; Goel et al., 2011; Chauhan et al., 2012; Huang et al., 2012; Karroum et al., 2012; Crokart et al., 2013; Huang et al., 2013; Cantelmo et al., 2016; Peterson et al., 2016; Martin et al., 2019; Mpekris et al., 2020). While the long-term effect of antiangiogenic treatments should lead to a deprivation of tumors from oxygen and nutrients, a transient normalization of the tumor vasculature (after the early pruning of immature vessels) occurs that can be exploited to potentiate the response to irradiation (by the reoxygenation of the tumor) (Ansiaux et al., 2005; Karroum et al., 2012; Crokart et al., 2013), to chemotherapy (by increasing the delivery of drugs) (Tong et al., 2004; Segers et al., 2006; Chauhan et al., 2012; Cantelmo et al., 2016) and to immunotherapy (Huang et al., 2012; Huang et al., 2013; Mpekris et al., 2020). The success of these combinations definitely requires the identification in individual tumors of the time window of increase in perfusion and/or oxygenation. It has been indeed demonstrated that the application of co-treatments (irradiation or administration of chemotherapeutic agent) outside the normalization window led to an absence of effect or even a decrease in therapeutic efficacy (Ansiaux et al., 2005; Segers et al., 2006).

Another strategy to increase the oxygen delivery without altering the perfusion is to promote the release of oxygen by hemoglobin (Hb). It has been shown that allosteric effectors (such as efaproxiral or myo-inositol trispyrophosphate) binding to Hb results in a decreased hemoglobin-oxygen (Hb-O_2_) affinity and an increased tumor oxygenation (Teicher et al., 1998; Khandelwal et al., 1999; Amorino et al., 2001; Hou et al., 2004; Hou et al., 2005; Suh et al., 2006; Hou et al., 2007; Scott et al., 2007; Aprahamian et al., 2011; Limani et al., 2016; Tran et al., 2019; Cao-Pham et al., 2020). The increases in regulated oxygen-releasing capacity of red blood cells has been shown to potentiate the response to irradiation (Teicher et al., 1998; Khandelwal et al., 1999; Amorino et al., 2001; Suh et al., 2006; Hou et al., 2007; Scott et al., 2007; Tran et al., 2019). It has been suggested that the beneficial aspect of allosteric effectors could also be mediated by the suppression of HIF-1α and to down-regulation of HIF-inducible genes such as VEGF (Aprahamian et al., 2011).

Additional oxygen can put in circulation by biocompatible perfluorochemical emulsions or nanoplatforms sometimes used as blood substitutes. These interventions are often combined with oxygen or carbogen breathing in order to potentiate anti-cancer therapy (Krafft, 2020). It has been described that these approaches with an increase blood oxygen-carrying capacity may lead to an increase in response to radiation therapy (Teicher et al., 1991; Koch et al., 2002; Song et al., 2017; Zhou et al., 2018), photodynamic therapy (Fingar et al., 1988; Cheng et al., 2015; Tang et al., 2017; Song et al., 2018; Wang et al., 2018; Hu et al., 2019; Kv et al., 2020; Fang et al., 2021), sonodynamic therapy (Zeng et al., 2020; Guo et al., 2021), chemotherapy (Teicher et al., 1987; Teic her et al., 1990; Teicher, 1994; Song et al., 2019; Wu et al., 2020), and immunotherapy (Jiang et al., 2021; Yang et al., 2021).

Compounds acting on tumor blood flow may also counteract fluctuating hypoxia. In this respect, nicotinamide (vitamin B3) has received particular attention (Horsman et al., 1988; Horsman et al., 1989; Chaplin et al., 1990; Kelleher and Vaupel, 1993; Siemann et al., 1994; Hill and Chaplin, 1995; Thomas et al., 1995; Powell et al., 1997; Baudelet et al., 2004). Nicotinamide improves microcirculatory function and the homogeneity of microregional blood flow (Powell et al., 1997). Nicotinamide was part of the large clinical trials together with carbogen breathing in the ARCON and BCON protocols (Kaanders et al., 1998; Kaanders et al., 2002; Hoskin et al., 2009; Janssens et al., 2012; Song et al., 2021). Alleviation of tumor acute hypoxia has also been reported using drugs that improve fluidity of red blood cells such as pentoxifylline (Lee et al., 1992) and flunarizine (Baudelet et al., 2004). The anti-angiogenic agent sunitinib during the early phase of normalization of the tumor vasculature has also been described to decrease cycling hypoxia (Matsumoto et al., 2011).

#### 2.1.2 Decreasing Tumor Hypoxia by Decreasing the Oxygen Consumption

The second major approach to increase tumor oxygenation is to modulate the metabolism (Gulledge and Dewhirst, 1996; Dewhirst et al., 2007; Danhier et al., 2013; Lin and Maity, 2015; Galle z et al., 2017; Dewhirst, 2018). The mathematical model described by Secomb suggested that a decrease in oxygen consumption should be much more efficient than an increase in oxygen delivery in order to alleviate tumor hypoxia (Secomb et al., 1995). In his computer simulations, he compared the effects of increasing blood pO_2_ or blood flow rate with a decrease in oxygen consumption rate. He showed that hypoxia can be abolished by a reduction in consumption rate by thirty percent, while it would require an increase in flow rate by a factor four or an increase in arterial pO_2_ by a factor of eleven or more (Secomb et al., 1995). Overall, this model suggested that decreasing oxygen consumption rate should be more effective than increasing blood flow or oxygen content to alleviate tumor hypoxia.

It is known that the individual basal metabolism is strongly dependent on hormones including thyroid hormones (Oppenheimer et al., 1987) with a potential impact in cancer management (Hercbergs, 1996). In tumor models, it has been demonstrated that the thyroid status was strongly impacting both tumor oxygenation (due to profound changes in tumor oxygen consumption rates) and response to irradiation. Tumors implanted in hypothyroid mice (treated with propylthiouracil) were much less hypoxic than in euthyroid or in hyperthyroid mice (treated with thyroxin) and, consequently, more responsive to irradiation (Jordan et al., 2007). General metabolic suppression (typically during mammalian hibernation) is in part under the control of hydrogen sulfide H_2_S, the last endogenous gas transmitter identified (Blackstone et al., 2005; Wagner et al., 2009). Taking benefit of the local acidic pH in tumors, it was found that the administration of the prodrug sodium hydrogenosulfide NaHS alleviated tumor hypoxia and radiosensitized tumors, an effect that was due to the inhibition of the mitochondrial respiration by tumor cells (De Preter et al., 2016b).

Besides general effect, other inhibitors of the tumor cell mitochondrial respiratory chain have been also described for their effect on tumor cell respiration, and tumor hypoxia with direct impact on the radiosensitivity. This effect has been described using non-steroidal anti-inflammatory drugs (piroxicam, diclofenac, indomethacin, NS398) (Crokart et al., 2005b), glucocorticoids (dexamethasone, hydrocortisone, prednisolone) (Crokart et al., 2007), metformin (Zannella et al., 2013), atovaquone (Ashton et al., 2016) and papaverine (Benej et al., 2018). Other compounds including some anti-angiogenic agents (Ansiaux et al., 2006b; Ansiaux et al., 2009; Crokart et al., 2013), MAPK inhibitors (Karroum et al., 2012) and EGFR inhibitor (Karroum et al., 2013) were also (unexpectedly) found to inhibit mitochondrial respiration in tumors cells and to potentiate radiation therapy.

Interestingly, cytotoxic agents may also contribute to an oxygen effect by decreasing the utilization of oxygen (Galle z et al., 2017). For example, it was shown that multiple factors contributed to the tumor reoxygenation induced by taxol and paclitaxel-loaded micelles (Milas et al., 1995a; Milas et al., 1995b; Danhier et al., 2012). It was found that the decrease in cell number affected the oxygen respiration (killed cells do not breath), an effect that added to the decrease in OCR affecting alive cells (Galle z et al., 2017). Paclitaxel-loaded micelles also induced an increase in tumor perfusion because of a decrease in the compression of venous vessels by cancer cells. As a consequence, the observed decrease in interstitial fluid pressure was correlated to an increase in tumor perfusion, and a consequent increase in oxygen delivery to tumor cells (Danhier et al., 2012). A comparable effect was observed after ranpirnase treatment, a cytotoxic amphibian ribonuclease (Kim et al., 2007; Lee et al., 2007). Of note, arsenic trioxide also induced an inhibition in tumor cell respiration at low non-cytotoxic concentration in pre-clinical tumor models, contributing to an increase in tumor oxygenation and a dramatic increase in tumor response to irradiation (Diepart et al., 2012).

Finally, it is essential to highlight the major interest for drugs that are releasing or stimulating the production of the free radical nitric oxide (NO). Nitric oxide presents a multi-faceted role in favoring the response to radiation therapy (Jordan et al., 2004; Sonveaux et al., 2009). First, nitric oxide is regulating mitochondrial respiration by virtue of reversible interactions with cytochrome c oxidase (complex IV in the mitochondrial respiratory chain) (Clementi et al., 1999). By inhibiting the oxygen utilization, the level of oxygen is increasing in solid tumors. Another important factor relies on the vasoactive properties of nitric oxide contributing to the increase in oxygen delivery. Finally, it should be emphasized that nitric oxide has intrinsic radiosensitizing properties comparable to oxygen through the fixation of DNA damages (Mitchell et al., 1993; Mitchell et al., 1996; Jordan et al., 2004). The release of nitric oxide can be achieved through the use of NO donors (Gallez et al., 1999a; Jordan et al., 2000; Jordan et al., 2003; Jordan et al., 2010), conversion of nitrite (Frérart et al., 2008), or stimulation of eNOS or iNOS (Jordan et al., 2002; Jordan et al., 2006a; Jiang et al., 2010). These multiple effects render nitric oxide release approaches highly efficient to potentiate radiation therapy.

### 2.2 Prodrugs Activated in Hypoxic Cells

**FIGURE 5:**
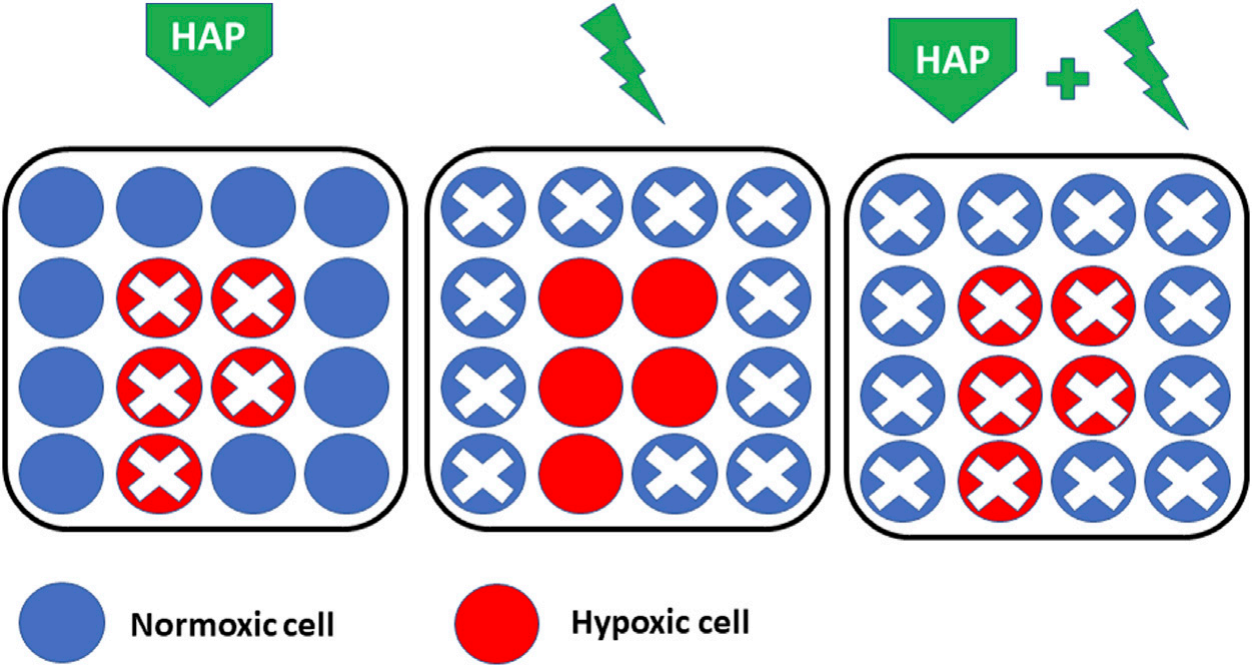
Rationale for combining HAP (hypoxia-activated prodrugs) with radiation therapy. HAPs are selectively killing hypoxic cells (Left) while radiation therapy is efficient in killing non-hypoxic cells (Middle). There is a major interest for combining both approaches for a maximal response (Right).

Hypoxia-activated prodrugs (HAPs) are bioreductive drugs that are selectively reduced by reductases under hypoxic conditions to form cytotoxic compounds that kill hypoxic tumor cells (Denny, 2000; Denny, 2010; Hunter et al., 2016; Phillips, 2016; Baran and Konopleva, 2017; Mistry et al., 2017; Jackson et al., 2019; Li et al., 2021a; Anduran et al., 2021; Li et al., 2021b; Codony and Tavassoli, 2021). This bio-reductive process is inhibited by oxygen preventing the complete reduction of the compound into its active form (Vilaplana-Lopera et al., 2021). In other words, HAPs should not be toxic for normal oxygenated tissues. As radiation therapy is very efficient in killing non-hypoxic cells and as HAPs are selectively killing hypoxic cells, there is a major interest for combining both approaches for a maximal response (Figure 5). As recently reviewed (Li et al., 2021b), several classes of HAPs have been developed and evaluated, including nitromidazoles, quinones, aliphatic and heteroaromatic N-oxides. A few illustrative examples of compounds that have received particular attention are presented hereafter.

Metronidazole and misonidazole were the first nitroaromatic drugs tested as potential radiosensitizers (Foster and Willson, 1973; Begg et al., 1974; Denekamp et al., 1974; Adams et al., 1976; McNally et al., 1978). While efficient in pre-clinical models, unexpected toxicity and absence of significant therapeutic benefit was observed in clinical trials combining these compounds with radiotherapy (Schwade et al., 1984; Coleman, 1985; Dische, 1985). A second generation of nitroimidazoles (including etanidazole, pimonidazole, and nimorazole) with improved pharmacokinetic properties and reduced toxicity was developed and evaluated clinically. While pimonidazole is nowadays largely used for assessing tumor hypoxia by immunohistochemical staining, pimonidazole and etanidazole did not demonstrate any clinical benefit when associated with radiation therapy (Dische et al., 1993; Eschwège et al., 1997; Urtasun et al., 1998). By contrast, nimorazole has been so far the only nitroimidazole that was demonstrated to improve the response in head and neck squamous cell carcinoma (HNSCC) treated by radiation therapy without increase in toxicity (Overgaard et al., 1998; Henk et al., 2003; Metwally et al., 2014; Hassan Metwally et al., 2015). Intriguingly, nimorazole has become a standard treatment for HNSCC in association with radiation therapy only in Denmark, and not in other countries. Evofosfamide (TH-302) is the last generation nitroimidazole compound linked to bromo-iso-phosphoramide mustard (Br-IPM). TH-302 is a substrate for cellular reductases that generate a radical anion through 1-electron reduction. Under normoxia, the free radical anions are oxidized back to the original prodrug. However, in hypoxia, the free radical anions are further reduced, leading to the release of Br-IPM (Duan et al., 2008; Li et al., 2021a; Li et al., 2021b). TH-302 has been found efficient in combination with irradiation in pre-clinical models (Lohse et al., 2016; Hajj et al., 2017; Nytko et al., 2017; Takakusagi et al., 2018a). TH-302 has been introduced in a series of clinical trials with several chemotherapeutic agents [see (Li et al., 2021a) for a review], but not yet together with radiation therapy.

Tirapazamine (TPZ, also known as SR-4233 or WIN 59075) is another HAP that can generate a reactive free radical through one-electron reduction (Zeman et al., 1986; Brown and Lemmon, 1990; Holden et al., 1992; Wang et al., 1992; Brown, 1993; Brown, 2000; Li et al., 2021b). TPZ was found highly efficient in increasing the effect of radiation *in vitro* and in pre-clinical models. The addition of TPZ to conventional chemoradiation protocols showed promising results in Phase II clinical trials in delaying recurrence and improving survival (Lee et al., 1998; Treat et al., 1998; Rischin et al., 2005). However, the Phase III trials did not confirm the benefit of using TPZ in association with radiation therapy (Williamson et al., 2005; Rischin et al., 2010), hampering the continuation of clinical trials. Of note, deficiencies in compliance with the initial protocols in these clinical trials were suggested to have contributed to the poor outcome observed in the association chemoradiation + TPZ (Peters et al., 2010).

Banoxantrone (AQ4N) is an aliphatic N-oxide that is activated under hypoxic conditions into AQ4 through a two-electron reduction mediated by cytochromes with a DNA affinity and cytotoxic potency about one thousand times higher compared to its prodrug (McKeown et al., 1995; Hejmadi et al., 1996). Banoxantrone has shown promises in pre-clinical models in association with radiation therapy and/or chemotherapy (Patterson et al., 2000; Patterson and McKeown, 2000; Gallagher et al., 2001). This compound has been used in Phase I clinical trial without providing any obvious benefit deserving further clinical trial so far (Steward et al., 2007; Albertella et al., 2008; Papadopoulos et al., 2008).

### 2.3 Inhibitors of Molecular Targets Involved in Hypoxic Cell Survival

As described previously, the activation of transcriptional factors HIFs regulates the expression of hundreds of genes involved in angiogenesis, invasion and metastasis, cell proliferation, cell survival, cell metabolism and tumor immunity (Semenza and Wang, 1992; Wang and Semenza, 1993; Semenza, 2000; Semenza, 2003; Loboda et al., 2010; Muz et al., 2015; Pugh and Ratcliffe, 2017; Albadari et al., 2019; Lee et al., 2020; Ban et al., 2021; Codony and Tavassoli, 2021; Ivan et al., 2021; Sebestyen et al., 2021). Consequently, the inhibition of HIF pathway could be useful in reversing hypoxia-induced effects and treating aggressive cancers (Semenza, 2006; Ban et al., 2021; Ivan et al., 2021; Sebestyen et al., 2021). HIF inhibitors may have different modes of action: they may interfere with HIF protein synthesis, they may promote HIF degradation and/or dimerization, and they may change DNA binding and transcriptional activity of HIF-1 and/or HIF-2 (Yu et al., 2017). Illustrative agents are described hereafter.

As HIFα mRNA is a limiting factor in the rate of protein synthesis, the antisense oligodeoxynucleotide EZN-2968 has been shown to downregulate the expression of HIF-1α protein in human biopsies of treated patients (Greenberger et al., 2008; Jeong et al., 2014). Topoisomerase 1 inhibitors (such as camptothecin, irinotecan, topotecan), were also shown to inhibit the expression of HIF-1α (Bertozzi et al., 2014). PX-478, a compound derived from melphalan by oxidation of the nitrogen mustard moiety, reduced the expression of HIF-1α mRNA and protein in human tumor xenografts, reduced the expression of HIF-1α target genes, and consequently decreased tumor progression and sensitized tumors to radiation therapy (Welsh et al., 2004; Jordan et al., 2005; Schwartz et al., 2009; Jacoby et al., 2010; Lee and Kim, 2011). It has been found that 2-methoxyoestradiol (that is also acting on microtubules) is also an inhibitor of the synthesis of HIF-1α and HIF-2α, and suppresses their transcriptional activity (Ma et al., 2014; Yu et al., 2017).

Other small molecules used in clinical trials such as the Hsp90 inhibitors geldanamycin and tanespimycin (17-AAG) have been shown to promote HIFα degradation (Isaacs et al., 2002; Mabjeesh et al., 2002; Neckers, 2002; Alqawi et al., 2006; Cao et al., 2008). Vorinostat, a histone deacetylase (HDAC) inhibitor is also suppressing the hypoxia signaling by promoting HIF degradation and modulating nuclear translocation of HIF-1α (Zhang et al., 2017). Several compounds also have been found to inhibit HIF dimerization such as acriflavine and PT2385 and were found to be active in a variety of cancer cell lines (Wong et al., 2012; Shay et al., 2014; Chen et al., 2016; Courtney et al., 2018; Montigaud et al., 2018; Courtney et al., 2020; Lequeux et al., 2021).

Another way to inhibit the HIF signaling cascade is to inhibit binding to DNA and interfere with the transcriptional activity. Echinomycin was shown to inhibit the binding of HIF-1 to the Hypoxia-Responsive Element (HRE) sequences (Kong et al., 2005; Vlaminck et al., 2007; Wang et al., 2014). The classical anti-cancer agents daunorubicin and doxorubicin (anthracyclines) also inhibit the binding of HIF-1 to the HRE sequences of the target genes (Kung et al., 2004; Yu et al., 2017). Of course, as these anthracyclines present pleiotropic effects, it is difficult to isolate the contribution of the HIF pathway to the tumor response to treatments.

It is also crucial to remind that HIF regulation could be mediated by several signaling pathways including NFκB, PI3K/AKT/mTOR, and MAPK/ERK. Inhibitors which target these upstream pathways not only impact their own targets but also the HIF pathway (DeBerardinis et al., 2008; Agani and Jiang, 2013; Aoki and Fujishita, 2017). Again, the isolation of sole contribution of HIF in tumor response is elusive, and the beneficial therapeutic observed is obviously coming from hitting multiple targets.

Hypoxia also activates unfolded protein response (UPR) signaling pathways in the endoplasmic reticulum (ER), which tries to restore ER homeostasis and function. Essentially, two main strategies can be used to target the UPR: 1) the inhibition of actors of the UPR (PERK, IRE1) so tumor cells can no longer adapt to the stressful environment thereby leading to cell death; 2) the exacerbation of the UPR stress so the already activated UPR is overloaded, thereby driving the cells towards the death pathway. (Healy et al., 2009; Di Fazio et al., 2012; Shapiro et al., 2016; Ojha and Amaravadi, 2017; Grandjean and Wiseman, 2020).

The inhibition of UPR components can be achieved through PERK inhibition (Healy et al., 2009; Axten, 2017; Ojha and Amaravadi, 2017). GSK2606414 and GSK2656157 are two PERK inhibitors that were found active in tumor models (Axten et al., 2012; Axten et al., 2013). More compounds have been developed to block the IRE1α-XBP1 pathway, including irestatin, toyocamycin, salicylaldimines, hydroxy-aryl-aldehyde, as illustrative examples (Li et al., 2011; Volkmann et al., 2011; Ri et al., 2012; Sanches et al., 2014; Ojha and Amaravadi, 2017). Available compounds that target IRE1α activity have shown potential for anti-cancer treatment in combination with other conventional chemotherapy (Mimura et al., 2012; Ri et al., 2012; Tang et al., 2014; Ojha and Amaravadi, 2017). On the side of drugs that exacerbate the UPR stress, thapsigargin and brefeldin A have been reported to activate all three branches of the UPR (Salles et al., 2004; Denmeade and Isaacs, 2005; Healy et al., 2009; Rajamahanty et al., 2010; Markouli et al., 2020).

## 3 Assessment of Tumor Hypoxia

The ideal clinical biomarker for assessing tumor hypoxia should combine the following characteristics: able to distinguish normoxia/hypoxia/anoxia/necrosis; able to distinguish between perfusion-related and diffusion-related hypoxia; able to reflect cellular oxygenation in preference to vascular oxygenation; being non-invasive; being applicable to any tumor site; being applicable in pre-clinical models and in patients; being simple to perform and non-toxic; allowing repeated measurements in longitudinal studies; providing maps or hypoxic regions; sensitive at pO_2_ relevant to tumor therapies; able to monitor the effect of treatments; predictive of the outcome. Despite intense research efforts in the development and validation of hypoxia biomarkers, we should admit that the optimal item does not (yet) exist. However, even with limitations, some approaches could be very useful to guide hypoxia-targeted interventions. In the next paragraphs, we will present a critical overview of different approaches to assess tumor hypoxia. Understanding their main characteristics will allow to define their potential interest as companion diagnostic for pharmacological interventions (see Table 1). The oxygen biomarkers may be categorized into methods providing real direct oxygen measurements and methods that are indirectly reflecting the presence of hypoxic regions.

**TABLE 1:** Key features of technologies for their use as hypoxia biomarkers and challenges for future validation as companion diagnostics.

Technology	Key features as hypoxia biomarkers	Challenges for future validation as companion diagnostics	References
Direct O_2_ measurements			
pO2 histography	• Quantitative pO2 assessments• Predictive of response to irradiation in the clinic • Not suitable for longitudinal studies• Not suitable for dose painting	• No more commercially available	Clark (1956), Clark and Lyons (1962), Kallinowski et al. (1990), Höckel et al. (1993a), Höckel et al. (1993b), Okunieff et al. (1993), Stone et al. (1993); Höckel et al. (1996), Fyles et al. (1998), Knocke et al. (1999), Dewhirst et al. (2000), Rudat et al. (2001), Nordsmark et al. (2005), Vaupel et al. (2007), Vaup el et al. (2021)
EPR oximetry (spectroscopy) with particulate sensors	• Quantitative estimates of pO2 (precision 1 mmHg)• Ideal for preclinical longitudinal studies for drugs modifying tumor oxygenation• Predictive of response to irradiation and to drug-induced modifications of hypoxia• Clinical studies only in a few centers worldwide• Limited to superficial tumors (1 cm depth)	• Technological development for in-depth measurements• Validation as predictive markers of response in the clinic	Gallez et al. (1999a), Jordan et al. (2000), Jordan et al. (2002), Pogue et al. (2002), Jordan et al. (2003), Pogue et al. (2003), Gallez et al. (2004), Hou et al. (2004), Crokart et al. (2005b), Hou et al. (2005), Ansiaux et al., (2006a), Jordan et al. (2006a), Ansiaux et al. (2006b); Martinive et al. (2006), Segers et al. (2006), Crokart et al. (2007), Khan et al. (2007), Frérart et al. (2008), Ansiaux et al. (2009), Ahmad and Kuppusamy (2010), Hou et al. (2010), Jordan et al. (2010), Segers et al. (2010), Karroum et al. (2012), Karroum et al. (2013), Matsumoto et al. (2014), Swartz et al. (2014), De Preter et al. (2016b), Swartz et al. (2016), Takakusagi et al. (2018b), Flood et al. (2018), Jeong et al. (2019), Flood et al. (2020), Gallez (2021), Schaner et al. (2021)
EPR oximetry (imaging) with soluble sensors	• Providing quantitative oxygen maps.• Predictive of response to irradiation and to drug-induced modifications of hypoxia• No clinical system available	• Technological developments of whole-body EPR imaging scanners.• Approval of oxygen sensors for human use• Validation as predictive markers of response in the clinic	Halpern et al. (1994), Gallez et al. (1996a), Ardenkjaer-Larsen et al. (1998), Elas et al. (2008), Charlier et al. (2009), Hyodo et al. (2009), Krishna et al. (2012), Elas et al. (2013), Khramtsov (2018), Chen et al. (2019), Epel et al. (2019), Nel et al. (2019), Sanzhaeva et al. (2020)
^19^F-relaxometry/imaging	• Providing quantitative oxygen maps• Predictive of response to irradiation and to drug-induced modifications of hypoxia• ^19^F-coils non-frequently used in the clinic	• Validation as predictive markers of response in the clinic	Fishman et al. (1989), Mason et al. (1991), Hees and Sotak (1993), Mason et al. (1993), Mason and Antich (1994), Mason et al. (1996), Hunjan et al. (1998), van der Sanden et al. (1999), Zhao et al. (2001), Zhao et al. (2002), Mason et al. (2003), Zhao et al. (2003), Nöth et al. (2004), Zhao et al. (2005), Bourke et al. (2007), Jordan et al. (2009), Diepart et al. (2011), Shi et al. (2013), Zhou et al. (2015), Chapelin et al. (2022)
Indirect O_2_ measurements			
R2*-MRI	• Endogenous contrast for mapping of blood oxygen saturation• No quantification of pO2• Predictive marker of response to modulation of blood oxygen saturation (oxygen/carbogen breathing)• Not predictive for modulation of tumor oxygen consumption	• Validation as predictive markers of response in the clinic	Karczmar et al. (1994), Robinson et al. (1995), Al-Hallaq et al. (2000), Howe et al. (2001), Baudelet and Gallez (2002), Rodrigues et al. (2004), Baudelet and Gallez (2005), Jordan et al. (2006b), Hoskin et al. (2007), McPhail and Robinson (2010), Liu et al. (2013), Tóth et al. (2013), Hallac et al. (2014), Kim et al. (2014), Gonçalves et al. (2015), Li et al. (2015), Cao-Pham et al. (2016a), Panek et al. (2017)
OE-MRI	• Endogenous contrast for induced changes in oxygenation• No quantification of pO2• Predictive marker of response in a few models	• Further validation as predictive markers in preclinical models• Validation as predictive markers of response in the clinic	Matsumoto et al. (2006), O'Connor et al. (2007), Winter et al. (2011), Linnik et al. (2014), O'Connor et al. (2016), Fan et al. (2017), White et al. (2016), Salem et al. (2019), Jordan et al. (2013a), Jordan et al. (2013b), Colliez et al. (2014), Colliez et al. (2015), Safronova et al. (2016), Cao-Pham et al. (2016b)
Combined R2*/OE-MRI	• Endogenous contrast for induced changes in oxygenation• No quantification of pO2• Potential markers of degree of hypoxia requiring further validation	• Further validation as predictive markers in preclinical models• Validation as predictive markers of response in the clinic	Cao-Pham et al. (2020), O'Connor et al. (2019), Hallac et al. (2014), Matsumoto et al. (2006), Winter et al. (2011), Remmele et al. (2013), Burrell et al. (2013)
DCE-MRI	• Quantitative estimates of blood flow/permeability• No quantification of pO2• Predictive for strategies modulating delivery• Not predictive for modulation of tumor oxygen consumption	• Validation as predictive markers of response in the clinic	Egeland et al. (2006), Vestvik et al. (2007), Benjaminsen et al. (2008); Egeland et al. (2008), Cho et al. (2009), Ellingsen et al. (2009), Newbold et al. (2009), Gulliksrud et al. (2010), Jordan and Gallez (2010), Gulliksrud et al. (2011), Øvrebø et al. (2011), Øvrebø et al. (2012), Borren et al. (2013), Ellingsen et al. (2014), Hallac et al. (2016), Hauge et al. (2017), Simoncic et al. (2017), Simonsen et al. (2018), Hou et al. (2019), Gaustad et al. (2020), Carmona-Bozo et al. (2021), Gaustad and Rofstad (2021), Liu et al. (2021)
^18^F-MISO	• Map of oxygen dependent trapping of nitroimidazoles• Relationship to tumor hypoxia debated• Predictive/unpredictive of response depending on models	• Require further preclinical validation for response to radiosensitizers• Validation as predictive markers of response in the clinic	Olive (1989), Koh et al. (1992), Lewis and Welch (2001), Gagel et al. (2004), The MICAD Research Team (2004), Eschmann et al. (2005), Cher et al. (2006), Rajendran et al. (2006), Lee et al. (2008), Lin et al. (2008), Spence et al. (2008), Lee et al. (2009), Choi et al. (2010), Mortensen et al. (2010), Hendrickson et al. (2011), Kikuchi et al. (2011), Chang et al. (2013), Sato et al. (2013), Kawai et al. (2014), Li et al. (2014), Lopci et al. (2014), Norikane et al. (2014), Fleming et al. (2015), Henriques de Figueiredo et al. (2015), Rajendran and Krohn (2015), Wiedenmann et al. (2015), Okamoto et al. (2016), Qiu et al. (2017); Welz et al. (2017), Xu et al., (2017), Sörensen et al. (2020), Zschaeck et al. (2020), Carle s et al. (2021)
^18^F-FAZA	• Map of oxygen dependent trapping of nitroimidazoles• Accumulation under 10 mmHg relevant for radioresistance• Predictive for radiation response• Predictive for nimorazole use as radiosensitizer• Potentially useful for dose painting	• Further validation as predictive markers of response in the clinic	Piert et al. (2005), Postema et al. (2009), Mortensen et al. (2011), Mortensen et al. (2012), Tran et al. (2012), Servagi-Vernat et al. (2014), Tran et al. (2014), Chang et al. (2015); Saga et al. (2015), Servagi-Vernat et al. (2015), Tran et al. (2015), Graves et al. (2016), Saga et al. (2016), Di Perri et al. (2017), Gammon et al. (2019), Vashisht Gopal et al. (2019), Elamir et al. (2021)
^18^F-HX4	• Map of oxygen dependent trapping of nitroimidazoles• Oxygen-dependence of trapping unknown• Predictive for radiation response in preclinical models• Potentially useful for dose painting	• Require further preclinical validation for response to radiosensitizers• Oxygen-dependence of trapping to be established• Validation as predictive markers of response in the clinic	Mahy et al. (2003), Dubois et al. (2011), Chen et al. (2012), Carlin et al. (2014), Peeters et al. (2015a), Peeters et al. (2015b), Zegers et al. (2016), De Bruycker et al. (2019a), De Bruycker et al. (2019b), Yu et al. (2019), Sanduleanu et al. (2020a), Sanduleanu et al. (2020b)
Cellular response to hypoxia			
CAIX/HIF-1α/…	• Requires biopsy• Classically interpreted as hypoxia biomarkers in immunohistochemistry• May be activated through other mechanisms than hypoxia• May be adapted for stratification in strategies targeting HIF• Non-adapted for longitudinal studies	• Further validation as predictive markers in preclinical models for strategies targeting HIF• Validation as predictive markers of response in the clinic	Semenza (2003), Vordermark and Brown (2003), Mayer et al. (2005a), Mayer et al. (2005b), Mayer et al. (2006), Muz et al. (2015)
Gene signature	• Requires biopsy• Preclinical validation established for most signatures• Prognostic value in many tumor types in the clinic• Predictive value in HNSCC, bladder cancer• Not adapted for longitudinal studies	• Validation as predictive markers of response in the clinic for most signatures	Fardin et al., (2010), Sørensen et al. (2010), Toustrup et al. (2011), Sta rmans et al. (2012), Toustrup et al. (2012), Eustace et al. (2013), Harris et al. (2015), Yang et al. (2017a), Yang et al. (2017b), Yang et al. (2018), Yang and West (2019); Fjeldbo et al. (2020)
CAIX/HIF-1α radioligands	• Non-invasive map of CAIX or HIF distribution in tumors• May be activated through other mechanisms than hypoxia	• Validation as predictive markers of response in preclinical models• Validation as predictive markers of response in the clinic	Kudo et al. (2009), Hoeben et al. (2010), Ueda et al. (2010), Kudo et al. (2011), Lau et al. (2016), Jia et al. (2019)

### 3.1 Direct Oxygen Measurements

The methods allowing direct oxygen measurements are those where a physicochemical property is directly dependent on the partial pressure of oxygen or the oxygen concentration in a tissue. In this category, we can find oxygen electrodes, optical measurements based on fluorescence quenching by oxygen, EPR oximetry and NMR fluorine relaxometry.

#### 3.1.1 Electrode Measurements

Micro-electrodes can be inserted directly into tissues to measure the pO_2_. These methods are derived from the seminal work of LC Clark to assess oxygen tension in the blood (Clark, 1956; Clark and Lyons, 1962). The reduction of oxygen at the cathode extremity generates a current proportional to the pO_2_. The Eppendorf^®^ pO_2_ histography system has a computerized driver that moves the electrode through the tissue minimizing compression and consumption of oxygen by the electrode (Kallinowski et al., 1990; Dewhirst et al., 2000). This system has been considered as the “gold standard” for assessing tumor oxygenation (Vaupel et al., 2007). This main achievement of this technology has been to definitely demonstrate that tumor hypoxia is a common feature of many solid tumors. Moreover, the method definitely established tumor hypoxia as a predictive marker of tumor outcome after different types of anti-cancer therapy (Höckel et al., 1993a; Höckel et al., 1993b; Okunieff et al., 1993; Stone et al., 1993; Höckel et al., 1996; Fyles et al., 1998; Knocke et al., 1999; Rudat et al., 2001; Nordsmark et al., 2005; Vaupel et al., 2007; Vaup el et al., 2021). In a critical evaluation of pO_2_ histography (Vaupel et al., 2007), P. Vaupel pointed as main advantages that the method provides absolute pO_2_ values with a precision around 1 mmHg within tissue micro-areas, provides several quantitative descriptive parameters and pO_2_ histograms within a tumor. However, the method is invasive and restricted to accessible tumors (such as head and neck, breast, or cervix cancer). While providing a distribution of pO_2_ along the electrode tracks, the method does not provide oxygen maps within the tumors (hypoxic regions cannot be excluded distant from the tracks). This means that pO_2_ histography may classify an individual tumor as likely hypoxic and may estimate its hypoxic fraction, but will not be useful for strategies of redistribution of radiation doses in treatment planning. Indeed, hypoxia-based dose painting is strongly dependent on the possibility to visualize and deliver appropriate radiation dose to hypoxic foci in tumors (Malinen et al., 2006; Grégoire et al., 2007; Sovik et al., 2007; Thorwarth et al., 2007; Lee and Le, 2008; Petit et al., 2009; Thorwarth and Alber, 2010; Bentzen and Gregoire, 2011; Toma-Dasu et al., 2012; Clausen et al., 2013; Geets et al., 2013; Hosk in, 2015; Servagi-Vernat et al., 2015; Welz et al., 2017; Gregoire et al., 2018). Another default of the method relies in its inability to differentiate between tumor and normal tissues and to discriminate measurements done in viable or necrotic regions (Vaupel et al., 2007). Finally, as the method is invasive, it is difficult to repeat measurements on the same tumor, for example to monitor the effect of treatments designed to alleviate tumor hypoxia in clinical longitudinal studies. At the pre-clinical level, the monitoring of drug effect using microelectrodes has been applied only acutely after application of a treatment or in different cohorts of tumors (treated vs. control) for chronic treatments (Horsman et al., 1989; Lee et al., 1993; Horsman et al., 1994; Hill and Chaplin, 1995; Horsman et al., 1998; Kelleher et al., 1998). Of note, the Eppendorf^®^ pO_2_ histograph is no more commercially available.

#### 3.1.2 Fiber-Optic Devices

Another way to assess tumor oxygenation is to use fiber-optic oxygen-sensing devices (such as the OxyLite^®^). In this system, photodiodes stimulate a fluorophore incorporated in a silicon polymer at the end of the tip, and the lifetime of the fluorescence is inversely proportional to the oxygen tension at the probe tip (Griffiths and Robinson, 1999). Compared to microelectrodes, the main advantage is that the measurement does not consume oxygen allowing the device to stay in place to monitor dynamic changes in oxygenation even in condition of extreme hypoxia. Pre-clinical studies have shown comparable measurements with microelectrodes, but differences in sampling volumes were noted (the OxyLite averages pO_2_ over a larger area than microelectrodes) (Griffiths and Robinson, 1999; Braun et al., 2001; Seddon et al., 2001). This device has been applied in a series of pre-clinical studies to monitor drug-induced changes in oxygenation (Jordan et al., 2002; Blackwell et al., 2003; Jorda n et al., 2003; Jordan et al., 2003; Wachsberger et al., 2005) and to assess the value of other hypoxia imaging modalities (Baudelet and Gallez, 2002; Demeure et al., 2002; Baudelet and Gallez, 2004; Zanzonico et al., 2004; Elas et al., 2006; Vikram et al., 2007; Jordan et al., 2009; Tran et al., 2012; Frank et al., 2015). As noted for the microelectrodes, the invasiveness, the need for repositioning the probe and the absence of spatial information limit their application for longitudinal studies. Of note, these probes do not possess CE or FDA regulatory approval for use in human subjects.

#### 3.1.3 Electron Paramagnetic Resonance Oximetry

**FIGURE 6:**
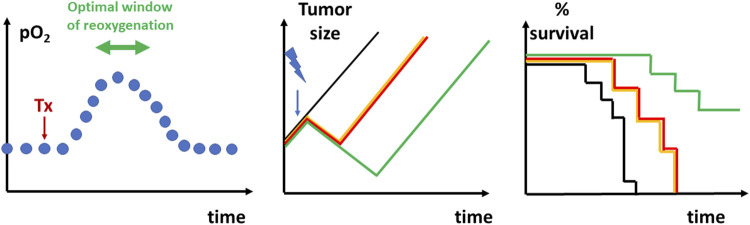
Graphical depiction of the identification of reoxygenation timing to radiosensitize tumors. *Left:* Longitudinal measurements of oxygenation (for example, using EPR oximetry) allows to define the window of reoxygenation after a pharmacological treatment (Tx). Depending of the treatment used and designed to alleviate tumor hypoxia, the window of reoxygenation may occur minutes, hours or days after initiation of a treatment. *Middle:* tumor regrowth delay experiment. Non-treated tumors (black) will progress regularly over time. Irradiated tumors (yellow) using suboptimal dose will typically present a transient decrease in tumor size due to the cytotoxic effect in a fraction of tumor cells before regrowing. The combination of a treatment together with irradiation administered outside the window of reoxygenation (red) will not lead to an increase in regrowth delay. The combination of the treatment with irradiation in the optimal timing of reoxygenation (green) is increasing the regrowth delay as more cells are killed by the irradiation. *Right*: Kaplan Meier curve representing the surviving fraction as a function of time depending on the treatment (colors represent the same groups than in the middle panel).

Quantitative assessments of tumor oxygenation can be obtained with EPR oximetry (spectroscopy and/or imaging) (Swartz and Clarkson, 1998; Gallez et al., 1999a; Subramanian et al., 2002; Dunn and Swartz, 2003; Gallez et al., 2004; Gallez and Swartz, 2004; Khan et al., 2007; Ahmad and Kuppusamy, 2010; Hyodo et al., 2010; Epel et al., 2011; Subramanian et al., 2012; Epel et al., 2014; Epel and Halpern, 2015; Gallez, 2021). EPR or equivalently ESR (Electron Spin Resonance) is a magnetic resonance method that detects species containing unpaired electron(s) (paramagnetic compounds). Molecular oxygen is paramagnetic, but no EPR spectra can be recorded from oxygen in tissues in physiological conditions. EPR oximetry methods are actually using the relaxing properties of oxygen which decreases the relaxation times of other paramagnetic compounds (Swartz and Clarkson, 1998; Gallez et al., 1999a; Dunn and Swartz, 2003; Gallez et al., 2004; Khan et al., 2007; Ahmad and Kuppusamy, 2010; Epel and Halpern, 2015; Gallez, 2021). T_1_ and T_2_ based measurements of paramagnetic reporters introduced in a biological system provide a direct indication of the oxygenation status (Gallez, 2021). Two classes of paramagnetic compounds can be used as oxygen reporters: soluble materials and insoluble particulate materials (Gallez et al., 2004; Gallez, 2021). Soluble materials include nitroxides (Halpern et al., 1994; Gallez et al., 1996a; Hyodo et al., 2009) and triarylmethyl (trityl) stable free radicals (Ardenkjaer-Larsen et al., 1998; Elas et al., 2006; Charlier et al., 2009; Krishna et al., 2012; Khramtsov, 2018; Chen et al., 2019; Nel et al., 2019; Sanzhaeva et al., 2020). The narrow EPR linewidth of trityl radicals is particularly suitable to obtain oxygen mapping with a high spatial resolution. The soluble EPR sensors present the inconvenience to be rapidly cleared from a tissue, requiring multiple administration if longitudinal oximetry studies are needed (Gallez, 2021). None of the soluble EPR reporters have been approved so far for clinical studies. Compared to soluble materials, particulate materials present two main advantages: they provide much more sensitive measurements of pO_2_ (variations of less than 1 mmHg can be detected) and, once introduced inside a tissue, they are reporting oxygenation from the same site over very long periods of time making them ideal probes for longitudinal studies (Gallez et al., 2004; Khan et al., 2007; Gallez, 2021). Particulate oxygen sensors include lithium phthalocyanine and derivatives (Liu et al., 1993; Ilangovan et al., 2002; Pandian et al., 2003), as well as paramagnetic carbon materials such as chars, coals, and carbon blacks (Vahidi et al., 1994; James et al., 1997; Jordan et al., 1998; Lan et al., 2004; Desmet et al., 2019). These oxygen paramagnetic reporters have been included in stable pharmaceutical suspensions or oxygen-permeable polymers to insure their biocompatibility (Gallez et al., 1996b; Gallez et al., 1998; Gallez et al., 1999b; Gallez and Mader, 2000; He et al., 2001; Charlier et al., 2004; Dinguizli et al., 2006; Meenakshisundaram et al., 2009a; Meenakshisundaram et al., 2009b; Meenakshisundaram et al., 2010; Hou et al., 2018). The unique capability of EPR oximetry to provide quantitative measurement of tumor oxygenation over time has been exploited in numerous preclinical studies [see (Gallez, 2021) for a review] after application of pharmacological challenges (Gallez et al., 1999a; Jordan et al., 2000; Jordan et al., 2002; Pogue et al., 2002; Jordan et al., 2003; Pogue et al., 2003; Hou et al., 2004; Jordan et al., 2004; Crokart et al., 2005b; Hou et al., 2005; Ansiaux et al., 2006a; Jordan et al., 2006a; Ansiaux et al., 2006b; Martinive et al., 2006; Segers et al., 2006; Crokart et al., 2007; Frérart et al., 2008; Ansiaux et al., 2009; Hou et al., 2010; Jordan et al., 2010; Segers et al., 2010; Matsumoto et al., 2011; Diepart et al., 2012; Karroum et al., 2012; Karroum et al., 2013; Matsumoto et al., 2014; De Preter et al., 2016b; Takakusagi et al., 2018b), carbogen/oxygen breathing challenges (Khan et al., 2009; Khan et al., 2010), or to measure the evolution of tumor oxygenation after irradiation (Goda et al., 1995; O'Hara et al., 1995; Goda et al., 1996; O'Hara et al., 1998; Sonveaux et al., 2002; Crokart et al., 2005a; Cron et al., 2005). EPR oxygen spectroscopy/imaging has been demonstrated as a valuable tool to predict the response to radiation therapy after alleviation of tumor hypoxia by most pharmacological challenges cited before. The identification of the temporal window of reoxygenation allows to propose a rationale for irradiation timing in order to optimize the response to treatment (Figure 6). EPR oxygen imaging also demonstrated its predictive value for tumor control according to tumor oxygenation level and radiation dose (Elas et al., 2008; Elas et al., 2013; Epel et al., 2019). So far, a limited number of studies have been applied in humans to measure by EPR the oxygen level in superficial tumors (Swartz et al., 2014; Swartz et al., 2016; Flood et al., 2018; Jeong et al., 2019; Flood et al., 2020; Schaner et al., 2021).

#### 3.1.4 Fluorine-NMR Relaxometry

^19^F relaxometry is a non-invasive magnetic resonance imaging (MRI) method providing quantitative maps of tumor oxygenation after the injection of a perfluorocarbon emulsion (Fishman et al., 1989; Mason et al., 1991; Mason et al., 1993; Mason and Antich, 1994; Mason et al., 1996; Hunjan et al., 1998; van der Sanden et al., 1999; Jordan et al., 2009; Shi et al., 2013). Calibration curves of the longitudinal relaxation rate (R_1_ or 1/T_1_) as a function of pO_2_ can be acquired for a given temperature and a given perfluorocarbon, and can be used to map tumor oxygenation quantitatively. This method has been used to measure the acute effect of pharmacological interventions or respiratory challenges designed to modulate tumor oxygenation (Hees and Sotak, 1993; Zhao et al., 2001; Zhao et al., 2002; Mason et al., 2003; Nöth et al., 2004; Zhao et al., 2005; Diepart et al., 2011; Zhou et al., 2015). Fluorine relaxometry has also been used to anticipate the response of tumors to irradiation (Zhao et al., 2003; Bourke et al., 2007; Chapelin et al., 2022) and to map spontaneous fluctuations in tumor oxygenation (cycling hypoxia) (Magat et al., 2010). For longitudinal studies, multiple injections are required and it has been shown the interest for using highly biocompatible perfluoro sensors (Mignion et al., 2013). Clinical applications of ^19^F MRI tumor oximetry measurement have not yet be implemented (Chapelin et al., 2022).

### 3.2 Indirect Oxygen Measurements

Numerous studies have been performed during the two last decades to develop and evaluate non-invasive imaging biomarkers of tumor hypoxia, including PET radiotracers and different sources of contrast in MRI. These developments have been comprehensively reviewed elsewhere (Horsman et al., 2012; Colliez et al., 2017; Price et al., 2013; Wijsman et al., 2013; Kelada and Carlson, 2014; Fleming et al., 2015; O'Connor et al., 2019; Busk et al., 2020; Huang et al., 2021; Matsumoto et al., 2021; Lopes et al., 2021; Padhani et al., 2007). Here, we summarize the principles of the principal approaches that have been developed, their added value and their limitations in the context of therapeutic guidance.

#### 3.2.1 PET Radiotracers of Tumor Hypoxia

**FIGURE 7:**
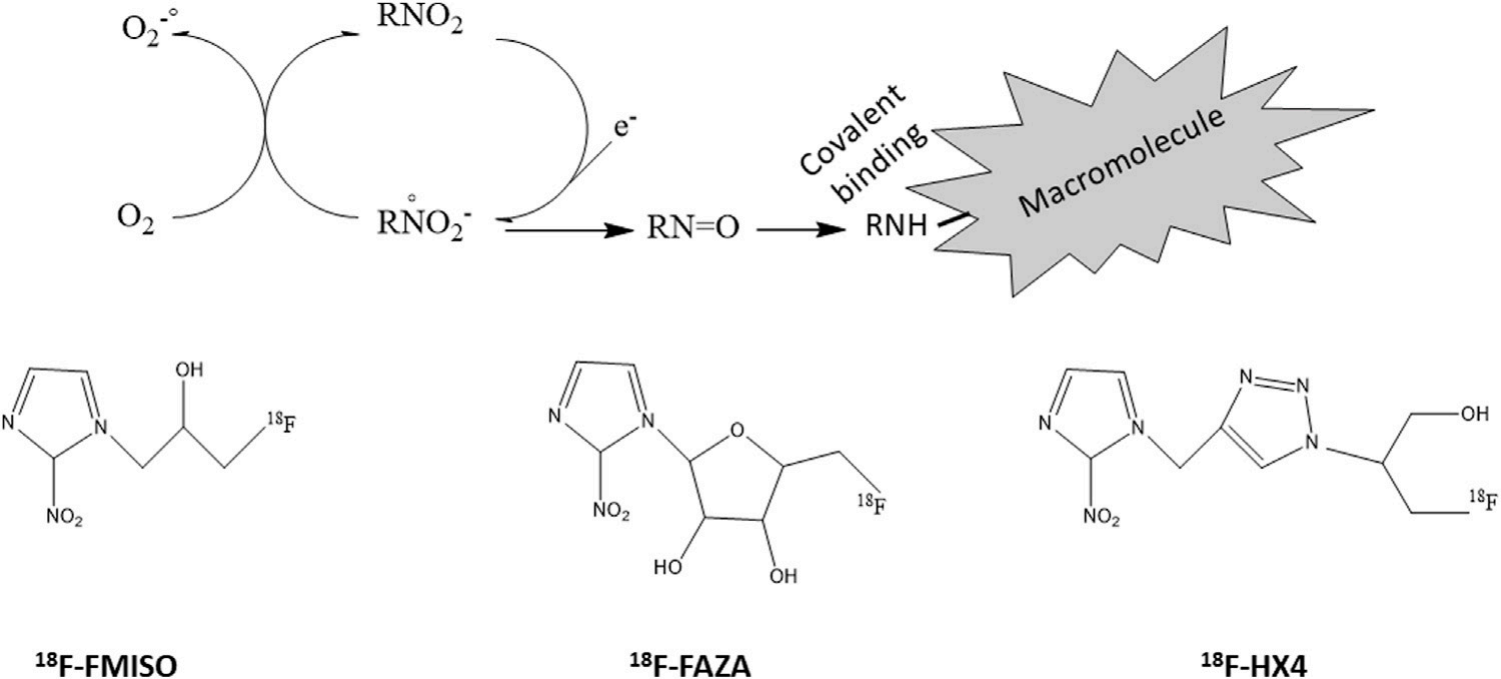
Radiolabelled nitroimidazoles. Top: Inside cells, nitroimidazoles (RNO_2_) are metabolized by reduction. This process is reversible under normoxic conditions leading to an equilibrium of the nitroimidazoles between the intra- and extracellular compartment. However, if cells are hypoxic, the radiotracer is further reduced and trapped by reacting with cellular macromolecules. Bottom: structures of commonly used radiolabeled nitroimidazoles (^18^F-FMISO, ^18^F-FAZA, ^18^F-HX4).

Hypoxia PET imaging requires the intravenous injection of a radiotracer (e.g., a nitroimidazole). While the initial distribution is flow dependent, the nitroimidazole is able to diffuse into cells and is reduced intracellularly. This process is reversible under normoxic conditions leading to an equilibrium of the nitroimidazoles between the intra- and extracellular compartment. However, if cells are hypoxic, the radiotracer is further reduced and trapped by reacting with cellular macromolecules (Figure 7) (Padhani et al., 2007; Kelada and Carlson, 2014; Fleming et al., 2015; Colliez et al., 2017). The reduction is under control of reductases that are only present in viable hypoxic cells. As a consequence, the accumulation of the hypoxic radiotracers is increased in hypoxic viable cells regions and not in necrotic cells. You should note that the process of accumulation of the hypoxia radiotracer is analog to the process of activation of prodrugs selectively killing hypoxic cells described in the section “2.2”. For PET imaging, the radiotracers have mostly been labeled with the ^18^F positron emitter (half-life: 110 min). Several ^18^F-nitroimidazoles have been developed, the most cited in the literature being ^18^F-FMISO, ^18^F-FAZA and ^18^F-HX4 (Figure 7).

^18^F-FMISO (^18^F-Fluoromisonidazole) is the most commonly used hypoxia radiotracer and was the first to be used in the clinic (Koh et al., 1992; Lewis and Welch, 2001; The MICAD Research Team, 2004; Wijsman et al., 2013; Li et al., 2014; Lopci et al., 2014; Fleming et al., 2015; Rajendran and Krohn, 2015; Xu et al., 2017). Due to its rather high lipophilicity, this compound easily crosses the cell membranes and is trapped in hypoxic cells. The cellular clearance of ^18^F-FMISO is rather slow in normoxic tissues, thereby hampering the contrast between normoxic tissues and moderate hypoxic tumor tissues. The tumor-to-background ratio (TBR) to define a hypoxic region is low (generally defined as 1.2–1.6) 2 h after the injection of ^18^F-FMISO (Koh et al., 1992; Li et al., 2014; Rajendran and Krohn, 2015). Despite its very large use, puzzling conflictual results were published in the literature regarding the relationship between its accumulation in tissues and the real level of hypoxia. For example, Gagel reported a significant correlation between tumor-to-muscle ratio of ^18^F-FMISO and parameters of hypoxic fraction in head and neck tumors as measured by pO_2_ histography (Gagel et al., 2004) while no correlation was found in another study (Mortensen et al., 2010). Xu et al. (2017) reviewed the few studies exploring the correlation between ^18^F-FMISO uptake and immunohistochemical expressions of HIF-1α and VEGF. ^18^F-FMISO PET uptake was correlated with HIF-1α expression in oral squamous cell carcinoma (Sato et al., 2013) while the correlation was weak in head and neck cancer (Norikane et al., 2014) and absent in gliomas (Cher et al., 2006; Spence et al., 2008; Kawai et al., 2014). Efforts have been made to assess the feasibility of using ^18^F-FMISO images for radiation therapy treatment planning and dose distribution according to the presence of hypoxic foci (Thorwarth et al., 2007; Lee et al., 2008; Lin et al., 2008; Choi et al., 2010; Thorwarth and Alber, 2010; Hendrickson et al., 2011; Chang et al., 2013; Henriques de Figueiredo et al., 2015; Qiu et al., 2017; Welz et al., 2017). These studies suggested that dose painting in hypoxic volumes was feasible. Several clinical studies in head and neck cancer observed that the uptake of ^18^F-FMISO as observed in PET imaging was predictive of the outcome after radiation therapy (Eschmann et al., 2005; Rajendran et al., 2006; Kikuchi et al., 2011; Sörensen et al., 2020; Zschaeck et al., 2020; Carle s et al., 2021) while another study did not observe such predictive value (Lee et al., 2009). Of note, the lack of standardized protocol to define hypoxia on the basis on ^18^F-FMISO uptake renders difficult the comparison between all these studies. Interestingly, the application of several ^18^F-FMISO PET acquisitions during the course of radiation therapy revealed a decrease in radiotracer uptake early after starting the treatment, an observation that is consistent with the reoxygenation of the tumors (Wiedenmann et al., 2015; Okamoto et al., 2016).

**FIGURE 8:**
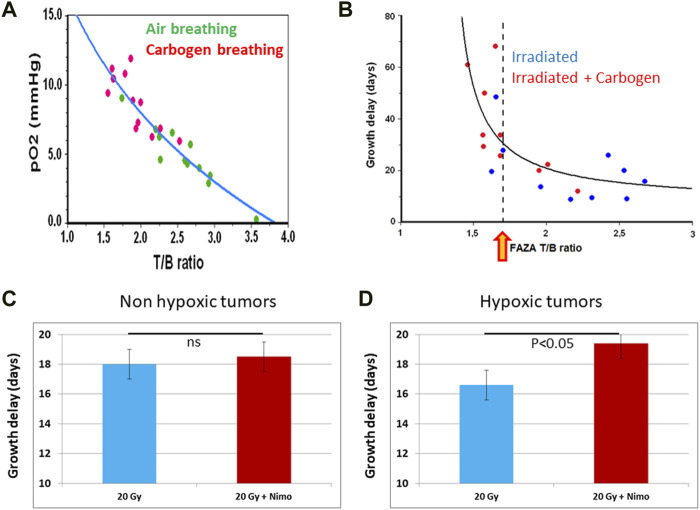
^18^F-FAZA as predictor of tumor response to radiation therapy. **(A)** *In vivo* calibration of ^18^F-FAZA tumor accumulation (measured by microPET) as a function of tumor pO_2_ (measured by EPR oximetry) in the same rhabdomyosarcoma tumors. **(B)** Growth time delay as a function of tumor uptake of ^18^F-FAZA (measured by microPET) in cohorts of animals breathing air or carbogen. The yellow arrow indicates a tumor-to-background ratio (T/B) corresponding to 10 mmHg (higher T/B means more hypoxic than this value while lower T/B means less hypoxic). **(C,D)** Value of ^18^F-FAZA tumor accumulation to predict the outcome of a treatment combining nimorazole together with irradiation. **(C)** for non-hypoxic tumors, no significant benefit (*p* > 0.05) was observed when tumors were treated by a combination of irradiation together with nimorazole (*n* = 7) compared to tumors treated with irradiation alone (*n* = 7). **(D)** for hypoxic tumors, a significant benefit was observed when tumors were treated by a combination of irradiation together with nimorazole (*n* = 9) compared to tumors treated with irradiation alone (*n* = 5). The figures are built with data from (Tran et al., 2012), (Tran et al., 2014), and (Tran et al., 2015).

^18^F-fluoroazomycin-arabinofuranoside (^18^F-FAZA) is another nitroimidazole that is more hydrophilic than ^18^F-FMISO (Piert et al., 2005; Postema et al., 2009). As a consequence, ^18^F-FAZA displays a faster clearance from the blood and the normal tissues than the more lipophilic ^18^F-FMISO. The delineation of tumor hypoxia with ^18^F-FAZA is obtained with a higher signal-to-noise ratio providing a better contrast imaging compared to ^18^F-FMISO. In a preclinical study, the prognostic value of hypoxia measured by ^18^F-FAZA or the Eppendorf oxygen electrode was assessed in a mammary carcinoma tumor model (Mortensen et al., 2011). ^18^F-FAZA PET showed that the accumulation of the radiotracer was predictive of response to irradiation similarly to the Eppendorf pO_2_ histography. In another preclinical study on rhabdomyosarcoma model, the ^18^F-FAZA uptake was compared to real pO_2_ values measured by EPR oximetry (Tran et al., 2012). A clear correlation between ^18^F-FAZA PET image intensities and tumor oxygenation was established: the accumulation of the radiotracer *in vivo* dramatically increased wen the pO_2_ was lower than 10 mmHg (Figure 8) (Tran et al., 2012). In another study, ^18^F-FAZA was found predictive of the response to radiation therapy (Tran et al., 2014). For 9L-gliomas, a significant correlation between ^18^F-FAZA tumor-to-background ratio (T/B) and tumor growth delay was found (Figure 8). In addition, carbogen breathing dramatically improved the tumor response to irradiation in this model. Rhabdomyosarcomas that were less responsive to hyperoxic challenge took advantage from dose escalation (Tran et al., 2014). ^18^F-FAZA PET was also found effective in guiding the use of nimorazole as radiosensitizer. The uptake of the radiotracer identified a subgroup of more hypoxic tumors that benefit from this combined treatment RT + nimorazole (Figure 8) (Tran et al., 2015). Pre-clinical studies also showed that ^18^F-FAZA PET could be used as a marker of response to treatments targeting tumor hypoxia trough the inhibition of mitochondrial respiration (Chang et al., 2015; Gammon et al., 2019; Vashisht Gopa l et al., 2019). In clinical studies, the treatment outcome was better for patients with non-hypoxic HNSCC tumors than for patients with hypoxic tumors as identified by ^18^F-FAZA PET (Mortensen et al., 2012; Graves et al., 2016; Saga et al., 2016; Zschaeck et al., 2020). In patients with advanced non-small-cell lung carcinomas (NSCLC), FAZA uptake in lymph nodes, but not in primary lesions, was predictive of treatment outcome (Saga et al., 2015). A PET study during radiation therapy revealed a decrease in ^18^F-FAZA uptake early after initiation of the treatment in HNSCCs (Servagi-Vernat et al., 2014), but not in NSCLCs (Di Perri et al., 2017). The feasibility of using ^18^F-FAZA PET for hypoxia-guided adaptive radiation dose escalation in hypoxic volumes has also been assessed in head and neck tumors and pancreatic cancer (Servagi-Vernat et al., 2015; Elamir et al., 2021).

^18^F-flortanidazole (^18^F-HX4) is another hydrophilic nitroimidazole that quickly clears from normoxic tissues allowing imaging 90 min after the radiotracer administration. This compound demonstrated promising preclinical and clinical results and a high TBR in hypoxic tumors (Sanduleanu et al., 2020a). The compound accumulates in regions with high hypoxic fraction as measured by pimonidazole (Dubois et al., 2011) and CAIX staining (Carlin et al., 2014). In a preclinical model of rhabdomyosarcoma, the uptake of ^18^F-HX4 was increased in animals breathing a gas with low oxygen content (7%) and decreased after carbogen/nicotinamide treatment (Dubois et al., 2011; Peeters et al., 2015a). However, contrarily to ^18^F-FAZA and EF5 for which tracer uptake was correlated to quantitative estimates of tumor oxygenation by microelectrodes or EPR oximetry (Mahy et al., 2003; Mortensen et al., 2011; Tran et al., 2012), the critical pO_2_ values at which ^18^F-HX4 is trapped in a tumor remain unknown. In a study where tumor rats were treated by irradiation, it was found that a higher ^18^F-HX4 uptake at baseline was associated with a worse prognosis (Yu et al., 2019). A preclinical study showed that the treatment efficacy of the HAP evofosfamide (TH-302) was dependent on tumor oxygenation as assessed by ^18^F-HX4: increasing the tumor oxygenation abolished the effect of evofosfamide, whereas enhancing the hypoxic fraction enlarged its therapeutic effect (Peeters et al., 2015b). Using metformin (an inhibitor of mitochondrial electron transport chain) to alleviate tumor hypoxia in NSCLC and colorectal cancer models, ^18^F-HX4 revealed a reduction in radiotracer uptake (De Bruycker et al., 2019a; De Bruycker et al., 2019b). The clinical trials with ^18^F-HX4 have been reviewed recently (Sanduleanu et al., 2020a): the assessment of tumor hypoxia with this radiotracer was favorable in NSCLCs, HNSCCs, and pancreatic cancer (Chen et al., 2012; Klaassen et al., 2015; van Elmpt et al., 2016; Zegers et al., 2016; Sanduleanu et al., 2020b). Establishing the clinical prognostic and predictive value will require more studies (Sanduleanu et al., 2020b).

We cannot conclude this section on PET radiotracers of tumor hypoxia without giving a few words on the continuously cited radiotracer ^60/62/64^Cu-ATSM (copper(II)diacetyl-bis(N4-methylthiosemicarbazone) using 2-deoxy-d-glucose). This compound has been and still continues to be suggested as a non-invasive PET biomarker of tumor hypoxia (Fujibayashi et al., 1997; Lewis et al., 1999; Chao et al., 2001; Dehdashti et al., 2003; Dehdashti et al., 2008; Holland et al., 2009; Sato et al., 2014; Pérès et al., 2019). However, its mechanism of retention in cells is highly controversial (Colombié et al., 2015; Liu et al., 2020). The initially proposed mechanism of retention in hypoxic cells is based on a pO_2_-dependence of one-electron reduction that is the discriminating factor which controls the reversibility of cellular uptake (Fujibayashi et al., 1997; Holland et al., 2009). However, the selective hypoxia dependence of the uptake and intracellular trapping has been questioned in numerous studies. It has been shown that Cu-ATSM accumulation was not dependent on hypoxic conditions in several tumor models (Yuan et al., 2006; Matsumoto et al., 2007). In addition, it was found a mismatch between Cu-ATSM accumulation and classical markers of hypoxia, for example Cu-ATSM showed the highest radiotracer uptake in regions with the low nitroimidazole uptake and CAIX staining (Carlin et al., 2014; Yuan et al., 2006; O'Don oghue et al., 2005; Valtorta et al., 2013; McCall et al., 2012; Hansen et al., 2012). It was also observed that tumor accumulation does not depend only on hypoxia but also on redox status and correlates with NADH/NADPH content, depends on MDR1 expression and on fatty acid synthase expression (Vave re and Lewis, 2008; Liu et al., 2009; Yoshii et al., 2012). For example, ^64^Cu-ATSM accumulated in cells with overreduced states due to mitochondrial dysfunction even under normoxia (Yoshii et al., 2012). It was also found that the uptake is cell line dependent (Burgman et al., 2005). Finally, it was also observed that the distribution of radiocopper from ^64^Cu-ATSM in tumors mirrors that of ^64^Cu-acetate suggesting that copper metabolism may also play a role in the mechanism of uptake (Hueting et al., 2014). Overall, despite its extensive use (likely due to its simple preparation and accessibility), Cu-ATSM should be considered as a non-reliable hypoxia biomarker considering the diversity of factors contributing to its retention in tissues.

#### 3.2.2 Magnetic Resonance ImagingUsing Endogenous Contrast

The endogenous contrast in MRI (without need of administration of a contrast agent) is mainly dependent on proton concentration, water motion and relaxation times (the time constants characterizing the return of the magnetization to its initial values after radiofrequency pulse excitation). Relaxations times include T_1_, T_2_ and T_2_*. The spin-lattice (or longitudinal) relaxation time T_1_ quantifies the rate of transfer of energy from the nuclear spin system to the neighboring molecules (the lattice). T_1_ is the time constant that characterizes the return kinetics of the magnetization along the longitudinal (z) axis. Spin-spin (or transverse) relaxation time T_2_ quantifies the decay rate of the magnetization within the xy plane (perpendicular to the applied magnetic field). After a 90° RF pulse, the nuclear spins become coherent in the transverse plane. The T_2_ characterizes the gradual loss in phase coherence. The combination of T_2_ relaxation and magnetic field inhomogeneity is referred to as the dephasing time or T_2_* (also called effective transverse relaxtion time). The inverse of relaxation times is referred to as relation rate R_1_ (=1/T_1_), R_2_ (=1/T_2_) and R_2_* (=1/T_2_*). The oxygen‐sensitive endogenous MRI contrasts R_1_ and R_2_* are potential imaging biomarkers of tumor hypoxia.

R_2_* uses the endogenous paramagnetic contrast agent deoxyhemoglobin (dHB) as a source of contrast that is at the origin of blood oxygen level-dependent (BOLD) contrast mechanism (Thulborn et al., 1982; Ogawa et al., 1990; Ogawa and Lee, 1990). dHb enhances the R_2_* effective transverse relaxation rates of water in blood and in the tissue surrounding the blood vessels. R_2_* is sensitive to the concentration of deoxyhemoglobin [dHb] per volume of tissue. A decrease in [dHb], due to an increase in blood oxygen saturation, results in a decrease in R_2_*. It should be emphasized that R_2_* is not only sensitive to the change in blood oxygenation through the change in [dHb], but is also sensitive to factors unrelated to the change in oxygenation, such as vascular volume, hematocrit, flow and vessel density (Howe et al., 2001; Baudelet and Gallez, 2005). In preclinical studies, T_2_* changes have been demonstrated to tackle changes in oxygenation levels during hyperoxic challenges (Karczmar et al., 1994; Robinson et al., 1995; Al-Hallaq et al., 2000; Hallac et al., 2014). Using simultaneous MRI measurements of the evolution of R_2_* together with direct oxygenation measurements by MR compatible fibre optics during oxygen/carbogen breathing challenges, no correlation has been established between R_2_* and absolute values of pO_2_: large differences in pO_2_ were observed for same R_2_* values, large differences in R_2_* were observed for same pO_2_ values (Baudelet and Gallez, 2002). Changes in R_2_* should therefore be considered as an indicator of changes in tumor oxygenation (measured in the vascular compartment) rather than a quantitative marker of the level of oxygenation. Studies compared R_2_* together with pimonidazole staining. A positive correlation was found in prostate cancer in patients (Hoskin et al., 2007) while an inverse correlation was observed rat mammary tumors (McPhail and Robinson, 2010). Attempts were also made to correlate R_2_* values with expression of HIF-1α: the mean R_2_* value correlated moderately with the level of HIF-1α in breast invasive ductal carcinoma (Liu et al., 2013), in human glioma (Tóth et al., 2013), but no correlation was found in renal carcinoma (Li et al., 2015). Using pharmacological manipulation of tumor oxygenation, it was found that the evolution of R_2_* was consistent with changes in tumor oxygenation after isosorbide dinitrate administration (Jordan et al., 2000) and the hemoglobin modifier ITTP (Cao-Pham et al., 2020). For the latter, the increase in R_2_* resulted from an increase in oxygen release from blood, inducing an increase in [dHb] (Cao-Pham et al., 2020). However, the evolution of R_2_* was not predictive of evolution of tumor oxygenation (as measured by EPR oximetry) after administration of inhibitors of oxygen consumption (insulin and AINS) (Jordan et al., 2006b) or after the RSR13 hemolobin modifier (Hou et al., 2004). The prognostic value of R_2_* as marker of response to irradiation was investigated in several preclinical studies. R_2_* was found predictive of outcome in rats exposed to carbogen challenges in GH3 prolactinomas (Rodrigues et al., 2004), in rat prostate tumors (Hallac et al., 2014), in 9L-gliomas but not in rhabdomyosarcoma (Cao-Pham et al., 2016a). The value of R_2_* as a predictor of therapeutic response was evaluated in thirthy cervical cancer patients undergoing concurrent chemoradiotherapy. This study showed that tumour R_2_* values were negatively correlated with final tumour size response, but not final tumor volume response (Kim et al., 2014). The application of serial BOLD MRI measurements has been used to provide non-invasive mapping of spontaneous fluctuations in tumor hypoxia (cycling hypoxia) with a high spatial and temporal resolution in tumor models (Baudelet et al., 2004; Baudelet et al., 2006; Gonçalves et al., 2015). The method demonstrated that treatment with cinnarizine or carbogen/nicotinamide led to a decrease in spontaneous fluctuations of oxygenation and blood flow (Baudelet et al., 2004). The method has also been translated into the clinic in head and neck cancer (Panek et al., 2017). The R_2_* fluctuation fraction was higher in the non-responding patient group, suggesting that the presence of such fluctuations may predict the outcome following treatment (Panek et al., 2017).

The dissolved paramagnetic molecular oxygen in tissue fluid and in blood plasma increases the spin lattice relaxation rate R_1_. It should be noted that the basal R_1_ of a tissue is influenced by many factors (i.e., components of the tissue including macromolecules and endogenous paramagnetic enzymes, mobility, … ) and therefore cannot be interpreted as marker of the tissue oxygenation (O'Connor et al., 2019). However, when switching the inhaled gas from air to an oxygen-enriched gas (100% oxygen or carbogen), the change in dissoved arterial oxygen may lead to a change in tumor oxygenation and consequently to a change in R_1_ (Matsumoto et al., 2006; O'Connor et al., 2007; Winter et al., 2011). This protocol is sometimes referred to as oxygen-enhanced MRI (OE-MRI) or tissue oxygenation level dependent (TOLD) contrast (O'Connor et al., 2019; Hallac et al., 2014; O'Connor et al., 2007). While changes in R_1_ in a voxel are resulting from changes in the vascular and tissue oxygen content, it is generally considered that the main effect is coming from the oxygen dissolved in the tissue, contrarily to changes in R_2_* that are reflecting changes in the vascular compartment. It was found in pre-clinical models that tumor regions with low response to OE-MRI were correlated to hypoxic regions evaluated by pimonidazole staining (Linnik et al., 2014; O'Connor et al., 2016; Fan et al., 2017). The response in OE-MRI was predictive of the response to radiation therapy (White et al., 2016). OE-MRI was also able to detect radiation-induced changes in tumor oxygenation in NSCLCs and glioma preclinical models as well as in NSCLCs in patients (Salem et al., 2019). To improve the sensitivity of oxygen enhanced R_1_ imaging, measurements of R1 in lipids was proposed to increase the sensitivity of the method (Jordan et al., 2013a; Jordan et al., 2013b). by exploiting the higher solubility of oxygen in lipids (as compared with water). The method provided highly sensitive and quantitative measurements of oxygenation in lipid-rich mammary cancer models (Colliez et al., 2014). However, while sensitive to changes in tissue oxygen content, the lipid R_1_ turned out to be no more sensitive than water R_1_ in tissues with lower lipid content, an observation that was done in preclinical models and in patients (Colliez et al., 2015; Cao-Pham et al., 2016b; Safronova et al., 2016).

ΔR_1_ and ΔR_2_* predictors of tumor oxygen evolution have been combined in a series of studies (Matsumoto et al., 2006; Winter et al., 2011; Burrell et al., 2013; Remmele et al., 2013; Hallac et al., 2014; Cao-Pham et al., 2020). Models were built thanks to combined measurements of ΔR_1_ and ΔR_2_* in order to discriminate different regional responses to oxygen/carbogen breathing challenges, including normoxia, mild hypoxia, severe hypoxia or vascular steal (Cao-Pham et al., 2020; O'Connor et al., 2019). While endogenous MRI contrast is particularly attractive due to non-invasiveness and large accessibility, clinical validation of ΔR_1_ and ΔR_2_* remains the main goal to achieve for considering them as reliable biomarkers for tumor-hypoxia targeted treatments.

#### 3.2.3 Magnetic Resonance Imaging With Exogenous Contrast

Dynamic contrast enhanced MRI (DCE-MRI) has been suggested to be a marker of tumor oxygenation in pre-clinical models (Egeland et al., 2006; Vestvik et al., 2007; Ege land et al., 2008; Ellingsen et al., 2009; Hauge et al., 2017; Hou et al., 2019; Gaustad et al., 2020) and in patients (Newbold et al., 2009; Simonsen et al., 2018; Gaustad and Rofstad, 2021; Liu et al., 2021). It has also been suggested to be a predictive marker of response to radiation therapy in pre-clinical models (Gulliksrud et al., 2011; Øvrebø et al., 2011; Øvrebø et al., 2012; Ellingsen et al., 2014; Hallac et al., 2016). It was also shown that hypoxia estimated by ^18^F-FMISO-PET correlated negatively with Ktrans from DCE-MRI in animal models, in head and neck cancers and in breast tumors, supporting the use of DCE-MRI to measure perfusion-driven hypoxia (Cho et al., 2009; Simoncic et al., 2017; Carmona-Bozo et al., 2021). However, several important limitations were also noted (Benjaminsen et al., 2008; Gulliksrud et al., 2010; Jordan and Gallez, 2010; Borren et al., 2013), including the absence of correlation of DCE-MRI parameters with the expression of HIF-1α and HIF-2α (Øvrebø et al., 2012). It should be emphasized that DCE-MRI takes only into account the perfusion without consideration to the oxygen consumption that plays also an important role in the establishment of tumor hypoxia. The interplay existing between tumor metabolism, oxygen consumption and oxygen delivery/availability may of course influence the occurrence of hypoxia. As a matter of fact, in a restrospective analysis evaluating the reliability of imaging biomarkers to predict changes in tumor oxygenation and improvement in tumor response to irradiation, DCE-MRI was predictive for strategies designed to increase the oxygen delivery through blood perfusion, but DCE-MRI was not predictive for strategies designed to modulate the tumor metabolism and oxygen consumption (Jordan and Gallez, 2010).

### 3.3 Biomarkers of the Cellular Response to Hypoxia

As discussed in section 1.2.1, cells exposed to hypoxia undergo a large variety of molecular responses, the predominant hypoxia-mediated intracellular signaling pathway being controlled by the transcription factors HIFs (Semenza and Wang, 1992; Wang and Semenza, 1993; Semenza, 2000; Harris, 2002; Semenza, 2003; Muz et al., 2015; Vaupel and Mayer, 2016; Pugh and Ratcliffe, 2017; Lee et al., 2020; Sørensen and Horsman, 2020). A possible way to identify hypoxic tumors is to focus not on oxygen itself but rather on the consequences of hypoxia exposure. Genes that are up- or downregulated in response to hypoxia reflect the hypoxic phenotype and therefore can provide an indirect measure of hypoxia. Their expression can be assessed in biopsies at the protein level using immunohistochemistry, or at the mRNA level, using gene expression arrays (Bussink et al., 2003; Vordermark and Brown, 2003; Mayer et al., 2006; Jubb et al., 2010; Toustrup et al., 2012; Adams et al., 2013; Harris et al., 2015; Swartz et al., 2015; Yang and West, 2019; Thiru thaneeswaran et al., 2021). Expression of HIF-1α and CA IX are classically analyzed by immunohistochemistry and used as markers of hypoxia. While there is no doubt that hypoxia leads to an induction of HIF-1α and CA IX *in vitro*, we should keep in mind that the expression of HIF-1α and downstream targets could also be achieved in a hypoxia-independent manner (Semenza, 2003; Vordermark and Brown, 2003; Mayer et al., 2005a; Mayer et al., 2005b; Mayer et al., 2006; Muz et al., 2015). It is also established that mutations of the von Hippel-Lindau gene result in normoxic stabilization of HIF-1α (Maxwell et al., 1999). Gene expression microarrays can also be used to determine a global transcriptional response to hypoxia. Genes found to be significantly upregulated, or exceeding a defined threshold from baseline normoxic expression, are typically grouped together and have been referred to as a “hypoxia gene expression signature” (Harris et al., 2015). As reviewed in (Toustrup et al., 2012; Harris et al., 2015; Yang and West, 2019), more than 30 hypoxia gene expression signatures have been published. As illustrative example, we may cite the development of a gene signature for hypoxia in head and neck cancer. A subset of genes upregulated under hypoxia were identified *in vitro* in several tumor cell lines (three oral carcinoma, one hypopharyngeal and one cervical carcinoma) (Sørensen et al., 2010), and then validated *in vivo* in xenograft HNSCC tumors by comparison with the FAZA nitroimidazole (Toustrup et al., 2011). The authors finally generated a gene expression classifier containing 15 genes (ADM, ALDOA, ANKRD37, BNIP3, BNIP3L, C3orf28, EGLN3, KCTD11, LOX, NDRG1, P4HA1, P4HA2, PDK1, PFKFB3, SLC2A1) that was validated in several hundreds of patients with HNSCC randomized for nimorazole or placebo in combination with radiotherapy (Toustrup et al., 2011). Tumors categorized as hypoxic on the basis of the classifier were associated with a significantly poorer clinical outcome than nonhypoxic tumors, and the outcome was equalized to the nonhypoxic tumors by addition of nimorazole to radiation therapy (Toustrup et al., 2011). Another 26-gene hypoxia signature has been found predictive for tumors in oropharynx treated by a combination of carbogen/nicotinamide together with accelerated radiation therapy (ARCON protocol) (Eustace et al., 2013). However, this signature was not predictive in bladder cancer treated by irradiation together with carbogen/nicotinamide (Eustace et al., 2013). Another 24-gene hypoxia signature has been found to be prognostic and predictive for muscle-invasive bladder cancer patients as the signature was able to identify patients likely to benefit from the addition of carbogen and nicotinamide to radiotherapy (Yang et al., 2017a). Other illustrative examples of hypoxia gene expression signatures include those found prognostic (but not yet tested for predictivity) in neuroblastomas (Fardin et al., 2010), prostate cancer (Yang et al., 2018), breast cancer (Sta rmans et al., 2012), and sarcomas (Yang et al., 2017b). Interestingly, a recent report suggested the benefit from the combination of imaging and gene expression signature to assess hypoxia-related treatment resistance and thereby enable more information about the disease before treatment-decision (Fjeldbo et al., 2020). Another interesting development relies on the use of miRNA signature. It has been recently shown that a 14-miRNA hypoxia signature can be used with an mRNA hypoxia signature to identify bladder cancer patients benefitting most from having carbogen and nicotinamide with radiotherapy (Khan et al., 2021).

Overall, these molecular markers of response to hypoxia coming from biopsies may definitely help in tumor characterization, prognostic and stratification in treatments targeting hypoxia. However, it is clear that, as for all non-imaging modalities, these markers cannot be used for dose painting in radiation treatment planning. As the method is invasive, it cannot be repeated in longitudinal studies for monitoring tumor hypoxia evolution and evaluate the efficacy of treatments designed to alleviate tumor hypoxia. In this regard, imaging biomarkers targeting CAIX and HIF have attracted attention. An antibody directed against CAIX and labeled with ^89^Zr (a positron emitter) ^89^Zr-labeled cG250-F(ab′)_2_ has been developed. In preclinical studies, this antibody accumulated in head and neck carcinoma xenografts with a spatial distribution correlated to CAIX expression and pimonidazole staining (Hoeben et al., 2010). Benzenesulfonamides radiolabeled with ^68^Ga selectively recognizing CAIX have also been developed and evaluated in pre-clinical tumor models (Lau et al., 2016). Another radiotracer ^18^F-CA IX-P1-4-10 has been developed: in preclinical studies, the distribution has been correlated with CA IX expression (Jia et al., 2019). It has been postulated that a probe containing the oxygen-dependent degradation domain (ODD) and inducing degradation in a similar manner as HIF-1α could be useful to evaluate HIF-1 activity *in vivo*. The protein transduction domain PTD and the ODD was fused with monomeric streptavidin (SAV) to produce a chimeric protein, PTD-ODD-SAV (POS). Radiolabeled biotin derivatives ^123^I-IBB and 18F-IBB were synthesized. POS was degraded in an oxygen-dependent manner and the accumulation of radiolabeled IBB-conjugated POS in the tumor was found to correlate with the HIF-1 activity (Kudo et al., 2009; Ueda et al., 2010; Kudo et al., 2011). There is a clear need for further validation in terms of prognostic and predictive values in preclinical models and in the clinic before considering these imaging biomarkers as potential companion diagnostic in hypoxia-targeted treatments. (Biomarkers Definitions Working Group, 2001).

## 4 Current Research Gaps and Potential Perspectives

The successful application of hypoxia-targeted interventions in patients is a challenging task that is will definitely benefit form the use of biomarkers used as companion diagnostics (Tatum et al., 2006; Parkinson et al., 2012; O'Connor et al., 2017; McAteer et al., 2021). As described earlier, there are existing numerous treatment approaches to counteract hypoxia or its consequences. On the other hand, numerous biomarkers have been described and are in the process of qualification or validation in preclinical and clinical trials. The biomarker may help at different levels in the pipeline of the research and development. In preclinical studies, the use of biomarker may help to confirm that the pharmacological agent is actually hitting its target and/or playing a role on the tumor microenvironment, may help in the selection of the lead compound among different candidates, may help to define the appropriate dosing and the time window of action. From preclinical studies, several potential companion diagnostics may be defined by cross-validation with other markers including invasive ones and post-mortem analyses of tissues. At early stages of clinical trials (Phase I/II), the value of companion diagnostics should be qualified as likely predictor of response. It will be then used for appropriate patient stratification in phase III clinical trials. If validated at this later phase, it should become an integrated part of the personalized medicine. Several key research gaps should be filled for future successful targeted-hypoxia interventions, including research needs in qualification/validation of biomarkers and appropriate coupling of companion diagnostics with the selected therapeutic target.

### 4.1 Hypoxia Biomarkers: Key Features and Challenges for Future Validation as Companion Diagnostics

**FIGURE 9:**
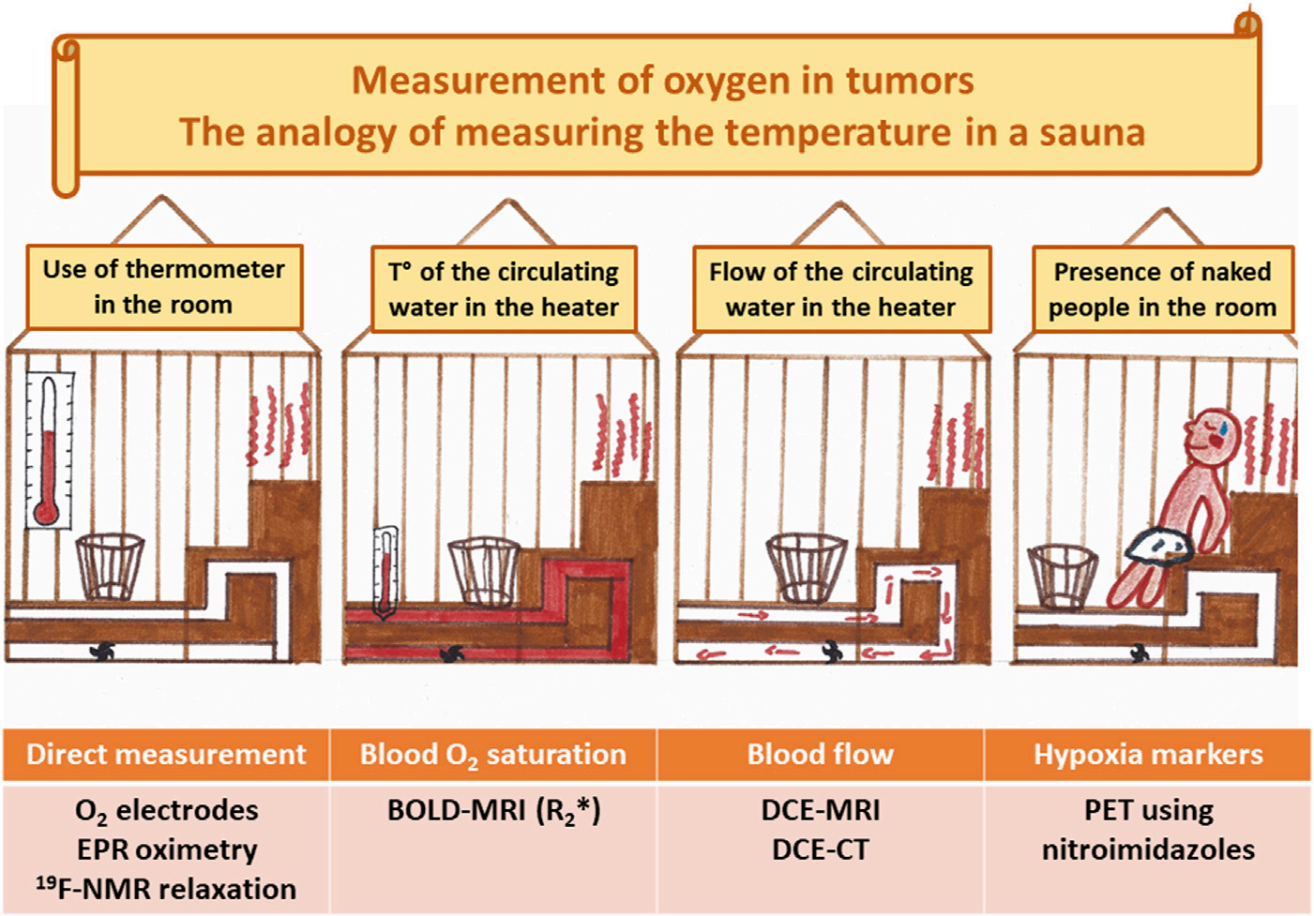
Comparison between measuring tumor oxygenation with hypoxia biomarkers and measuring temperature in a sauna. The direct way to measure the temperature is to use a thermometer. Translated in the hypoxia world, it corresponds to direct measurements of tissue oxygenation (pO_2_ histography, optic fibres, EPR oximetry, ^19^F-relaxometry). Another approach is to measure the temperature of the circulating water in the heater. Caution: we do not know if the door is open. This approach is analog to the measurements done using the oxygen blood saturation (R_2_* or BOLD-MRI). It provides information on the oxygen delivery through the blood. Another approach is to measure the flow of the circulating water in the heater. Caution: we do not know if the door is open and if the circulating water is hot. It is comparable with DCE-MRI. The method only considers the delivery, no information is provided regarding the oxygenation. Finally, we may look for the presence of naked (or almost) people in the room. While we do not know the temperature, their presence likely means a hot temperature inside the sauna. This situation is analog to the accumulation of nitroimidazoles inside hypoxic cells. We do not know the real oxygenation of the tissue, but it is likely hypoxic.

In our description regarding possible ways to assess hypoxia, we have started by stating that the ideal hypoxia biomarker does not exist yet. That does not mean that existing biomarkers are not helpful. In this regard, some comparison may be helpful to understand their merits and flaws for their convenient application as companion diagnostic. Let’s compare the measurement of hypoxia with the measurement of temperature in a sauna (Figure 9). The most obvious way to measure the temperature is to use a thermometer directly in the sauna. Translated in the hypoxia world, it corresponds to direct measurements of tissue oxygenation (pO_2_ histography, optic fibres, EPR oximetry, ^19^F-relaxometry). However, most direct methods are invasive and/or not (or not easily) accessible in the clinic. Another approach is to measure the temperature of the circulating water in the heater. It may help, but we should be cautious as we do not know if the door is open. In other words, we have an information on the delivery of calories, but not on their consumption and the real temperature observed in the sauna remains unknown. This approach is quite analog to the measurements done using the oxygen blood saturation (R_2_* or BOLD-MRI). While not providing information on oxygen *per se*, it may be helpful for checking the effect of strategies designed to modify the blood oxygen saturation (oxygen or carbogen breathing) or release from hemoglobin. We may also measure the flow of the circulating water in the heater. It may help, but we should be cautious as we do not know if the door is open and if the circulating water is hot. It is comparable with DCE-MRI. As the method only considers the delivery, no information is provided regarding the oxygenation. However, it could be helpful to assess the effect of treatments designed to modulate the tumor perfusion. Finally, we may look for the presence of naked (or almost) people in the room. While we do not know the temperature, their presence likely means a hot temperature inside the sauna. This situation is analog to the accumulation of nitroimidazoles inside hypoxic cells. We do not know the real oxygenation of the tissue, but it is likely that related compounds that are trapped by the same mechanisms (such as the radiosensitizer nimorazole) can also accumulate in the same cells.

In the Table 1, we have summarized the current status and the challenges for future validation of the different biomarkers as companion diagnostics.

### 4.2 Questions/Answers on the Selection of the Hypoxia Biomarker

Knowing that no hypoxic biomarker is ideal, the choice of a biomarker should be closely related to the scientific question to be addressed. That means that a biomarker may change or evolve during the research progression and phase of development. In the next paragraphs, we provide a pragmatic/practical approach and critical guidance for defining the couple “biomarker/hypoxic targeted intervention” in diverse situations. These suggestions are of course based on the present status of development and qualification of technologies. There is no doubt that the choice will evolve as the technologies and their validation progress. Besides the guidance advices, the purpose is to stimulate the critical thinking before using an approach.

*I am radiation oncologist. Is this tumor hypoxic?* Quantitative mesurements are not likely available except in specialized centers that still possess the pO_2_ histography Eppendorf^®^ system that can provide absolute pO_2_ measurements. Clinical EPR oxygen measurements are still at an early stage of development. The most straightforward, even indirect, way to get the answer is to request CAIX and HIF-1α from biopsies and, if possible, hypoxic gene expression and/or miRNA signatures. However, we should keep in mind that these biomarkers are not fully validated for the purpose. These biomarkers suffer from possible HIF-1α activation through a hypoxia-independent pathway in some circumstances. The addition of a PET scan with nitroimidazoles should be considered. The value of ^18^F-FMISO is debated regarding conflictual cross validation with other traditional hypoxia biomarkers while ^18^F-FAZA has been shown to accumulate in tumors under 10 mmHg, a meaningful level regarding radiosensitivity/radioresistance.

*I am radiation oncologist. Which biomarker is adapted for dose painting?* Knowing that a tumor is hypoxic thanks immunochemistry or gene signature assays is obviously not sufficient for therapeutic guidance in this context. Irradiation treatment adaptation strongly relies on the availability of reliable tumor oxygen mapping. EPR imaging is the only method that provides quantitative oxygen maps, but the technology is still under development and in validation at the preclinical stage. PET images obtained from radiolabeled nitroimidazoles can potentially be useful for radiation therapy treatment planning and dose distribution according to the presence of hypoxic regions. While treatment planning has been established with different tracers, only ^18^F-FAZA has been tested *in vivo* for comparison between radiotracer trapping and real pO_2_ values, showing a dramatic increase in accumulation under 10 mmHg. At the present stage of development, MRI approaches (OE-MRI, R_2_*-MRI, DCE-MRI) have not been validated for this purpose.

*I am radiation oncologist. Which biomarker is adapted for selecting a radiosensitizing approach?* The response is dependent on the radiosensitizing approach. Let’s first consider HAP (hypoxia activated prodrugs)*.* This approach requires a go/no go decision for its use in a specific patient based on the presence of hypoxia. Phase III clinical trials have demonstrated that hypoxic gene expression signature is useful as predictor of response for the association nimorazole together with radiation therapy for treating head and neck cancer. The translation to other types of tumor and/other gene signature obviously requires a similar validation. An alternative is to consider PET scan with radiolabeled nitroimidazoles for this purpose. As most HAPs are trapped inside tumor cells using a mechanism similar to nitroimidazoles, this may be considered as a potentially useful biomarker for this application, of course requiring validation according to tumor type and nitroimidazole that will be used. For approaches using interventions designed to alleviate hypoxia by oxygen/carbogen breathing challenges, OE-MRI and R_2_*-MRI could be used as pharmodynamic biomarker of response in individual patients. However, as these MRI methods do not provide access to tissue oxygenation levels, it has to be combined with information provided by immunohistochemistry staining and/or gene signature regarding hypoxia at the basal level. The advantage of MRI is to provide in a simple challenge a dynamic evolution of parameters related to oxygen in vascular and tissue compartments, as well as the presence or absence of response to a specific challenge.

*I develop a pharmacological intervention to alleviate tumor hypoxia. Which biomarker is useful during pre-clinical development and clinical development?* At a preclinical stage, direct quantitative measurements can be applied in small animals. To increase statistical power and decrease the number of animals included in the studies, preference should be given to non-invasive longitudinal studies on the same tumor. Dynamic real-time changes in tumor oxygenation from the same sites can be achieved with EPR oximetry. A few centers offer this possibility and EPR systems adapted for the purpose are now commercially available (O2M technologies, Chicago, IL, United States; Novilet, Poznan, Poland; Bruker, Rheinstetten, Germany). In a small cohort of animals, the appropriate dosing and identification of time window of effect can be quickly identified and extended to a series of tumor models. Based on these generated robust data, other biomarkers could be tested as the ambition is to have a natural prolongation up to clinical trials. The other biomarkers should be selected on the basis of the mechanism of action of the pharmacological intervention. For strategies based on changes in oxygen delivery through an increase in blood oxygen saturation or change in hemoglobin saturation curve, the recommendation is to look for the value of combined OE-MRI and R_2_*-MRI. For strategies designed to facilitate the tumor perfusion, DCE-MRI could be added in the evaluation. For strategies designed to decrease the oxygen consumption, OE-MRI, R_2_*-MRI and DCE-MRI are not valuable methods, and ^17^O-MRI could be considered. For strategies that are rapidly changing tumor oxygenation, it is unlikely that PET with nitroimidazoles will gain any value as dynamic changes in oxygenation cannot be tackled considering the long distribution and elimination time of the radiotracers. Except for EPR, all these methods do not provide real pO_2_ measurements. For appropriate stratification in the clinic, combining these predictors together with immunohistochemistry and/or gene signature may be valuable to assess tumor hypoxia before treatment.

*I develop a new strategy targeting HIF or downstream signaling cascades. Which biomarker is useful during clinical development?* The identification of potential candidates for a treatment targeting HIF can be based on immunohistochemistry from biopsies revealing the overexpression of HIF-1α or using a gene signature focused of HIF activated genes. Ideally, after validation at the preclinical stage and in early clinical trials (Phase I/II), it could be used as a stratification tool for the enrollment of patients in Phase III, and later when applied routinely in the clinic. The problem is that the biomarker based on biopsy is a snapshot taken only before treatment, and nothing will be known on the possible evolution of expression of HIF over time. This is an appeal to further develop radiotracers targeting HIF that could be used for longitudinal studies. Of course, this will require full validation as predictive marker of response to be considered as potential companion diagnostic in HIF-targeted treatments.
